# Functional connectivity and home range inferred at a microgeographic landscape genetics scale in a desert‐dwelling rodent

**DOI:** 10.1002/ece3.4762

**Published:** 2018-12-11

**Authors:** Alejandro Flores‐Manzanero, Madisson A. Luna‐Bárcenas, Rodney J. Dyer, Ella Vázquez‐Domínguez

**Affiliations:** ^1^ Departamento de Ecología de la Biodiversidad, Instituto de Ecología Universidad Nacional Autónoma de México Ciudad de México México; ^2^ Posgrado en Ciencias Biológicas Universidad Nacional Autónoma de México Ciudad de México México; ^3^ Department of Biology and Center for Environmental Studies Virginia Commonwealth University Richmond Virginia

**Keywords:** desert ecosystems, *Dipodomys merriami*, functional connectivity, landscape genetics, Merriam’s kangaroo rat, resistance surfaces

## Abstract

Gene flow in animals is limited or facilitated by different features within the landscape matrix they inhabit. The landscape representation in landscape genetics (LG) is traditionally modeled as resistance surfaces (RS), where novel optimization approaches are needed for assigning resistance values that adequately avoid subjectivity. Also, desert ecosystems and mammals are scarcely represented in LG studies. We addressed these issues by evaluating, at a microgeographic scale, the effect of landscape features on functional connectivity of the desert‐dwelling *Dipodomys merriami*. We characterized genetic diversity and structure with microsatellites loci, estimated home ranges and movement of individuals using telemetry—one of the first with rodents, generated a set of individual and composite environmental surfaces based on hypotheses of variables influencing movement, and assessed how these variables relate to individual‐based gene flow. Genetic diversity and structure results evidenced a family‐induced pattern driven by first‐order‐related individuals, notably determining landscape genetic inferences. The vegetation cover and soil resistance optimized surface (NDVI) were the best‐supported model and a significant predictor of individual genetic distance, followed by humidity and NDVI+humidity. Based on an accurate definition of thematic resolution, we also showed that vegetation is better represented as continuously (vs. categorically) distributed. Hence, with a nonsubjective optimization framework for RS and telemetry, we were able to describe that vegetation cover, soil texture, and climatic variables influence *D. merriami*'s functional connectivity at a microgeographic scale, patterns we could further explain based on the home range, habitat use, and activity observed between sexes. We describe the relationship between environmental features and some aspects of *D. merriami*‘s behavior and physiology.

## 1 INTRODUCTION

Since the term was coined in 2003, landscape genetics (LG) has been a constantly growing field that combines population genetics, landscape ecology, and spatial analytical techniques to quantify the effects that the landscape has on microevolutionary processes (Balkenhol, Cushman, Storfer, & Waits, 2015; Manel, Schwartz, Luikart, & Taberlet, 2003; Storfer, Murphy, Spear, Holderegger, & Waits, 2010). One such process is the movement of genes among populations (i.e., gene flow) that, in animal species, is primarily determined by the dispersal of individuals, at different scales, across the landscape (Reding, Cushman, Gosselink, & Clark, 2013). A variety of features within the landscape matrix may limit or facilitate the movement and gene flow of individuals (structural and functional connectivity). Landscape representation in LG has been traditionally modeled as resistance surfaces (RS), which can be defined as a spatial layer that assigns a value to each of a set of landscape features, and where values denote the degree to which a feature limits or facilitates connectivity across the landscape. Resistance surfaces can be viewed as hypotheses of the relationship between landscape variables and gene flow (Spear, Balkenhol, Fortin, McRae, & Scribner, 2010; Spear, Cushman, & McRae, 2015; Zeller, McGarigal, & Whiteley, 2012).

A common method for developing RS is the parameterization of resistance values based on expert opinion (Murray et al., 2009) to test alternative costs ratios (e.g., 2:1, 10:1, 100:1), in which one variable is hypothesized to always facilitate (e.g., habitat value = 1) and the other to restrict movement at different orders of magnitude (nonhabitat value = 2, 10, 100). This approach has been amply applied in natural systems (Howell, Delgado, & Scribner, 2017; Spear & Storfer, 2008, 2010). Simulation studies have demonstrated that the value of nonhabitat relative to habitat is key for detecting an effect on gene flow, in which a higher contrast in the costs ratios will make this relationship more detectable (Cushman, Shirk, & Landguth, 2013; Jaquiéry, Broquet, Hirzel, Yearsley, & Perrin, 2011). More recently, LG studies using this parameterization approach have also implemented mirror‐like cost ratios, by assigning high cost values not only to nonhabitat but also to habitat patches (i.e., 10:1, 2:1, 1:2, 1:10), which eliminates the potential bias of only assigning increasing cost values to variables considered to restrict gene flow (see Hohnen et al., 2016). Notwithstanding, expert opinion RS development is based on arbitrary costs with no consensus about the values assigned for the cost ratios, frequently assuming a linear relationship between continuous variables and genetic distances, which may not always be the case (Spear et al., 2010, 2015). Because accurate inferences about these relationships are dependent on both, the correct representation of the landscape and of the values of landscape variables, we require methods for assigning resistance values that adequately avoid subjectivity (Richardson, Brady, Wang, & Spear, 2016; Spear et al., 2010). A recent proposal is to simultaneously evaluate multiple surfaces and a wide range of cost values, without making any assumptions about their relationship with genetic distances. Specifically, Peterman, Connette, Semlitsch, and Eggert (2014) and Peterman (2018) used Ricker and monomolecular equations to transform data, in combination with linear mixed‐effects models and genetic algorithms. This RS optimization framework does not make a priori assumptions regarding the scale and direction of the resistance relationship, allowing to perform both the optimization of categorical surfaces and the simultaneous optimization of multiple RS.

Landscape genetic studies have focused mostly on terrestrial animals and temperate forest ecosystems (Dyer, 2015; Storfer et al., 2010), whereas tropical and specially desert ecosystems are significantly lacking. Deserts represent one of the most extended ecosystems on Earth—nearly a third of the globe—exhibiting environmental characteristics like extreme temperatures and low precipitation regimes, which result in low net primary productivity and scarce vegetation cover (Whitford, 2002). For such reasons, desert ecosystems may seem to harbor little heterogeneity and, consequently, no significant role of landscape features in determining evolutionary processes like gene flow; the latter is likely associated with their underrepresentation in LG literature (Storfer et al., 2010). However, desert landscapes are truly heterogeneous ecosystems where, among others, vegetation patches retain rainfall, resulting in a banded vegetation pattern tightly linked to topography and soil type (Grünberger, 2004; Ludwig, Wilcox, Breshears, Tongway, & Imeson, 2005). Notably, of the few LG studies done on desert ecosystems, fewer have been performed for mammal species, including big‐sized and long dispersing mammals such as the bighorn sheep (*Ovis canadensis nelsoni*; Creech, Epps, Monello, & Wehausen, 2014a, 2014b), a small carnivore (*Bassariscus astutus*; Lonsinger, Schweizer, Pollinger, Wayne, & Roemer, 2015), and a desert rodent (*Dipodomys spectabilis*; Cosentino, Schooley, Bestelmeyer, McCarthy, & Sierzega, 2015), in which some landscape features like vegetation and elevation influenced gene flow.

Rodents are considered a key ecological component of deserts due to the fundamental role they play for the structure and dynamics of these ecosystems, especially by the dispersal of seeds and soil removal (Brown & Heske, 1990a, 1990b). In addition, rodents have been proposed as ideal for conducting LG research (Waits, Cushman, & Spear, 2015) because of their small body size, short generation times, and limited dispersal abilities. Accordingly, LG studies have focused on different rodent species living in a wide range of environments, including tropical dry forests (spiny pocket mouse, *Liomys pictus*; Garrido‐Garduño, Téllez‐Valdés, Manel, & Vázquez‐Domínguez, 2015), savannas of South Africa (Natal multimammate mouse, *Mastomys natalensis*; Russo, Sole, Barbato, von Bramann, & Bruford, 2016), subantarctic forests and Patagonian steppes (Long‐tailed pygmy rice rat, *Oligoryzomys longicaudatus*; Ortiz et al., 2017), and even in urbanized areas (White‐footed mouse, *Peromyscus leucopus*, Munshi‐South, 2012; and Norway rats, *Rattus norvegicus*, Gardner‐Santana et al., 2009). Interestingly, vegetation has been underlined as a variable facilitating gene flow in some of those systems, including the canopy cover across New York City for *P. leucopus* (Munshi‐South, 2012), tropical dry forest corridors in *L. pictus* (Garrido‐Garduño et al., 2015), and forest patches in chipmunks (*Tamias striatus*) inhabiting agroecosystems (Kierepka, Anderson, Swihart, & Rhodes, 2016), whereas in *O. longicaudatus,* none of multiple landscape features (lakes, rivers, urban settlements, roads) facilitated dispersal. Notably, studies regarding the importance of elements promoting gene flow on rodents from desert environments are significantly lacking.

One of the most conspicuous desert rodents is kangaroo rats (genus *Dipodomys*, family Heteromyidae). In particular, the Merriam's kangaroo rat *Dipodomys merriami* is one of the smallest species in the genus, with a distribution that encompasses the desert region of southwestern United States and northern Mexico. It is a burrow‐dwelling, nocturnal rodent characterized by a long tail with a tuft of hair at the tip, large rear legs for bipedal locomotion, and with sexual dimorphism where males are larger than females. It is considered solitary and territorial, characterized by dispersing adult males (mean of 60 m) and phylopatric females that make parental investment (Behrends, Daly, & Wilson, 1986; Randall, 1993; Zeng & Brown, 1987). Their mating system is polygynandrous, where matings occur mainly between close neighbors (Randall, 1993) during February and July. They show a mean survival of 3.5 years, with two litters per year and an average of three young per litter (Zeng & Brown, 1987), feeding commonly on mesquite seeds (*Prosopis sp*.) and always building their burrows under bushes like mesquite and creosote (*Larrea tridentata*) (Murrieta‐Galindo & Cuatle‐García, 2016; Reynolds, 1958).

As a desert organism, *D. merriami* is challenged by the low productivity, extreme temperatures, and low precipitation regimes of its habitat. Indeed, *D. merriami* has shown to be highly sensitive to food shortage under controlled conditions (Banta, 2003), in agreement with a desertic environment where food is scarce, and thus rendering mesquite (and other desert plants) key for its survival (Reynolds, 1958). Moreover, *D. merriami*'s daily activity (e.g., foraging, dispersal, breeding) is affected by ambient temperature (29–34ºC; French, 1993; Banta, 2003) and relative humidity (40%; Reynolds, 1958; Frank, 1988; Walsberg, 2000). Although some information about the natural history and ecology of *D. merriami* exists (e.g., Behrends et al., 1986; Zeng & Brown, 1987; Randall, 1993), the effect of landscape and environmental features on genetic structure and connectivity has not been evaluated.

The space where an animal species performs all its activities (e.g., feeding, reproduction, dispersal, avoiding predators) defines what is known as home range (Burt, 1943); a home range represents an interplay between the environment and an animal's understanding of that environment (i.e., its cognitive map) (Powell & Mitchell, 2012). The selection of a place to live among different alternatives available (habitat selection), tightly linked to the home range, is a hierarchical process that involves both innate and learned behaviors (Partridge, 1978). Recent advances in telemetry, geographical positioning systems, and home range estimators have facilitated the description of habitat selection patterns and home ranges in wild species, at diverse micro‐ and macroenvironmental scales; allowing, in turn, the understanding of organismal movement, how and why animals use specific resources, and other elements that determine fitness and survival (Kernohan, Gitzen, & Millspaugh, 2001; Demšar et al., 2015). Importantly, combining knowledge on habitat use, behavior, and population genetics can significantly contribute to the interpretation of functional connectivity in landscape genetics (Portanier et al., 2018).

We here apply the optimization framework developed by Peterman et al. (2014) and Peterman (2018) to evaluate, at a microgeographic scale, the effect of landscape features on gene flow of *Dipodomys merriami* in northern Mexico. For that purpose, we first characterized genetic diversity levels and population genetic structure; next, we generated a set of environmental surfaces based on hypotheses of variables influencing the species movement, to finally assess how these variables relate to individual‐based genetic differentiation. In order to obtain estimates of the species habitat use and home range that will inform our genetic results, we evaluated movement of individuals with telemetry. Given that *D. merriami* is tightly linked to shrub cover and occurrence of fine‐sized soils for protection against predators, feeding, and burrow construction, we expected that (a) females and males will mostly overlap home ranges; (b) the presence of vegetation associated with sandy and gravel soils would be a key predictor of gene flow, while uncovered habitat (i.e., bare soil and/or rocky landscapes) would restrict movement; (c) environmental variables (e.g., temperature, humidity) will significantly influence patterns of functional connectivity in this desert‐dwelling rodent.

## 2 MATERIALS AND METHODS

### 2.1 Study site and sampling

**Figure 1:**
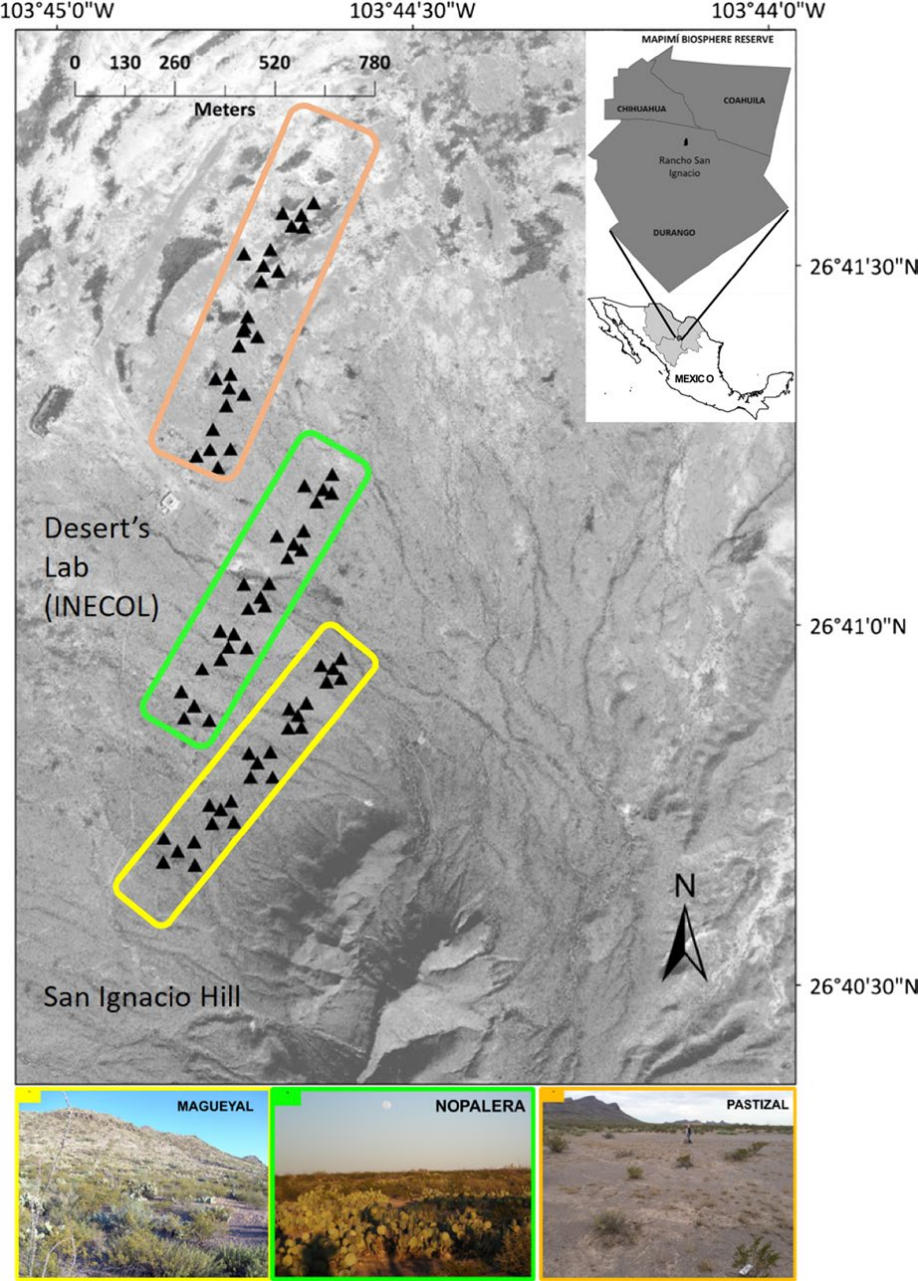
Study site and sampling locations for *Dipodomys merriami* individuals on the Mapimí Biosphere Reserve, Mexico. Black triangles correspond to the trapping webs (most external points), which are projected on a grayscale aerial picture of the study site, where gray shades are vegetation patches. The three vegetation transects are depicted, separated by approximately 200 m, where we placed five trapping webs per transect, separated 50 m from each other. Each web consisted of four 25‐m lines radiating from a central point (often a *Dipodomys merriami* burrow). The dominant vegetation per transect is depicted with different colors and a corresponding photograph (at the bottom). For more detail, see Supporting Information Figure S1 in Supplementary Information

The Chihuahuan Desert is one of the largest deserts in North America, known as the Bolsón de Mapimí in Mexico, where our study was conducted at the Mapimí Biosphere Reserve (MBR), a protected natural area and a Man and Biosphere (MAB‐UNESCO) site (Montaña, 1988) (Figure 1). The MBR extends 342,387 ha, representative of the arid ecosystems from northern Mexico that harbors a high endemism and species richness (Conanp, 2006). The climate is dry (mean annual precipitation: 271 mm, mean annual temperature: 20.8°C, rainy season from July to October and elevation range: 1,100–1,650 m; Montaña & Breimer, 1988; Conanp, 2006). The vegetation is dominated by shrubby species like mesquite (*Prosopis glandulosa*) and creosote bush (*Larrea tridentata*). Despite the zone is relatively flat, the landscape has been described as a toposequence, on the base of landforms, soils and vegetation units organized along an elevation gradient (Grünberger, 2004; Montaña & Breimer, 1988) and vegetation units: “magueyal” (predominating *Agave sp*. and *L. tridentata*), “nopalera” (*Opuntia sp*.), and “pastizal” (*Prosopis sp*.) (Martínez & Morello, 1977) (Figure 1). Sampling was performed from 31 May to 11 June 2015, using a modification of the trapping web sampling method (Anderson, Burnham, White, & Otis, 1983) in order to include the most environmental heterogeneity across the scale of the study; importantly, this trapping web has proven to be effective for capturing *D. merriami* in the MBR (Aragón, Castillo, & Garza, 2002; Hernández et al., 2005). Briefly, the web design consists of lines (*L*) of equal length radiating from a chosen center point, each line with live traps (*T*) separated between them at certain intervals (Figure 1, Supporting Information Figure S1 in the Appendix S1). Our sampling method was as follows: (a) We established vegetation units based on visual inspection at the base of the San Ignacio Hill slope, along which we set three vegetation transects (hereafter transects), separated by approximately 200 m; (b) we placed five trapping webs in each transect, separated 50 m from each other, where each web consisted of four 25‐m lines radiating from a central point (often a *D. merriami*'s burrow). Sherman live traps (7.6 x 7.6 x 33 cm) were placed along each line, separated by 5 m, and baited with a mixture of rolled oats, peanut butter, and vanilla extract. Additionally, two more traps were placed at 1 m distance from each burrow entrance; thus, each web consisted of 22 traps (Figure 1; Supporting Information Figure S1 in the Appendix S1). Trapped individuals were sexed and measured; a tissue sample (earpunches) was taken for genetic analysis and stored in labeled Eppendorf tubes with 96% ethanol. All individuals were released at the sampling site. Procedures were conducted according to the American Society of Mammalogists guidelines for use of wild mammal species (Sikes, 2016) and with the corresponding collecting permits.

### 2.2 Population genetic analyses

We extracted DNA from tissue samples to test 14 fluorescently labeled microsatellite primers developed for *D. spectabilis*, from which we successfully amplified nine with *D. merriami* (see the Supporting Information Appendix S1 and Table S1 for more information).

We tested for deviation from Hardy–Weinberg equilibrium (HWE) and linkage disequilibrium (LD) across the entire population using Fisher's exact test (1,000 batches and 100 000 iterations) with genepop v.6 (Rousset, 2008). The value was adjusted for multiple comparisons using a Bonferroni correction (Rice, 1989). We checked for the presence of null alleles and stuttering with microchecker (Van Oosterhout, Hutchinson, Wills, & Shipley, 2004). Genetic diversity levels were assessed by estimating number of alleles, number of effective alleles, unbiased expected and observed heterozygosity, and *F*_IS_ with gstudio in R (Dyer, 2014), while relatedness between individuals was assessed with ML‐Relate (Kalinowski, Wagner, & Taper, 2006).

We inferred population structure with two approaches, the Bayesian clustering method implemented in structure v.2.3.4 (Pritchard, Stephens, & Donnelly, 2000) and the discriminant analysis of principal components (DAPC; Jombart, Devillard, & Balloux, 2010). For structure, we tested from *K* = 1 to *K* = 13 genetically distinct clusters with 20 replicates for each *K*, based on an initial burn‐in of 2 x 10^5^ followed by 5 x 10^5^ Monte Carlo Markov chain iterations and an admixture model with correlated allele frequencies. In addition, we incorporated the sampling information (*locprior* option) to the model by considering each trapping web as a different location. The posterior probability lnP(K) (Pritchard et al., 2000) and ∆K (Evanno, Regnaut, & Goudet, 2005), implemented in Structure Harvester (Earl & vonHold, 2012), were used for identifying the most likely number of clusters. Although both methods are suitable for analyzing spatially continuous data, the structure algorithm considers population genetics models (Pritchard et al., 2000). Because some natural systems can violate some model‐assumptions which in turn may lead to errors in the assignment of individuals to populations, we performed an additional multivariate, nonmodel‐based approach (DAPC), using adegenet 2.1.0 in R (Jombart et al., 2010), following a two‐stage procedure: First, a principal component analysis (PCA) is performed with the genetic data; then, the principal components (PCs) of the PCA are processed with a linear discriminant analysis (LDA). Since DAPC relies on the determination of predefined groups that are often unknown, they must be identified a priori. Thus, we first inferred genetic clusters by running the *K*‐means clustering algorithm, from *K* = 1 to *K* = 13, with *find.clusters*. For this step, we retained all 76 PCs based on the recommendation of keeping all (or at least 80%) of the information (Jombart & Collins, 2015). Next, based on the lowest Bayesian information criterion (BIC) value, we determined the optimal number of clusters, with which we ran the *dapc* function. Unlike the *K*‐means clustering algorithm, DAPC benefits from not using too many PCs. Finally, to determine the number of PCs and also avoid overfitting during discrimination, we used the *optim.a.score* and *xvalDapc* functions; the *scatter* function was used to build an ordination plot with the results. Finally, we also estimated pairwise *F*_ST_ values between genetic clusters inferred by each method with hierfstat 0.04–22 in R (Goudet, 2015).

Considering that evidence of population structure can be found when family members are included in a sample, as would be the case for *D. merriami*, even when such structure is absent (Anderson & Dunham, 2008), we identified first‐order relatives (full siblings, FS; and parent–offspring, PO) based on the previous relatedness analysis (Kalinowski et al., 2006). Next, we removed one individual of each dyad and ran both analyses again with the same parameters for this unrelated dataset. For DAPC analysis, we retained 59 PCs in the *find.clusters* function with this dataset (see Results). This procedure has shown to improve accuracy on estimates of genetic structure (Rodríguez‐Ramilo & Wang, 2012), particularly when conducting landscape genetics studies (Peterman et al., 2014; Ruiz‐Lopez et al., 2016).

Finally, genetic dissimilarity at the individual level (i.e., between all pairs of individuals) was estimated as the proportion of shared alleles (*D*_PS_) with adegenet in R (Jombart et al., 2016). We chose *D*_PS_ because it makes no biological assumptions and can be used for populations at any level of ploidy or inbreeding; it has also proved to be an adequate metric for performing individual‐based genetic distance estimates (Shirk, Landguth, & Cushman, 2017). To test for isolation by distance, we performed a simple Mantel test between genetic (*D*_PS_) and Euclidean distances. Additionally, a Mantel correlogram was estimated based on 50 m classes considering the mean dispersal distance reported for *D. merriami* (Zeng & Brown, 1987) and our own home range results. Spearman correlation significance for both analyses was based on 10,000 permutations. Euclidean distances were estimated with gstudio (Dyer, 2014), while the Mantel test and correlogram were calculated with ecodist (Goslee & Urban, 2007), both in R.

### 2.3 Landscape data

Since most of the freely available environmental data is at coarse scales (e.g., WorldClim ca. 1 km^2^ resolution; Hijmans, Cameron, Parra, Jones, & Jarvis, 2005), we generated a set of environmental surfaces at our study spatial scale which were hypothesized to affect survival or movement of *D. merriami*: humidity (as a proxy for precipitation at a very local scale), temperature, elevation, plant cover (hereafter vegetation), and soil. Humidity and temperature data were collected using HOBO Data Loggers (Pro v2 and UX100–003, ONSET Computer Corporation), and elevation was obtained with a GPS (Garmin). These three variables were measured at each trap location and at the center of the trapping web (i.e., 21 points per trapping web and 105 by transect), for a total of 315 points; the values for the entire study area were obtained by the krigging interpolation method (Holdaway, 1996) with ArcGIS v10.2.1.

We used two approaches to create the vegetation and soil surfaces. First, we calculated the normalized difference vegetation index (NDVI, Rouse, Haas, Deering, Schell & Harlan, 1974) using ERDAS IMAGINE v13.0 from a Landsat 8 image (ID: LC80300412015222LGN00, 30 m resolution, available free at http://glovis.usgs.gov). According to the U. S. Geological Survey ( https://phenology.cr.usgs.gov/index.php), values for this index range from −1 to 1, based on the different behavior of vegetation and soils in the red and near‐infrared spectral regions: areas of barren rock or sand usually show very low NDVI values (0.1 or less), while sparse vegetation such as grasslands or shrubs may result in moderate NDVI values (approximately 0.2 to 0.5). Second, we performed a supervised classification based on our field data using the high‐resolution (approximately 1.3 m) imagery from Google Earth. This surface corresponded to a binary classification (presence/absence) of vegetation (hereafter Feature surface).

Using lower‐resolution imagery to characterize land cover can lead to incorrect or misleading evaluations of connectivity if not verified on the field (Zeller, Nijhawan, Salom‐Pérez, Potosme, & Hines, 2011), and on the other hand, some satellite bands are not available in Google Earth imagery, thus lacking information about certain landscape features (Boyle et al., 2014). Accordingly, due to the fine scale of this study, we chose to use both Landsat 8 (lower‐resolution) and Google Earth (higher‐resolution) data to more adequately discern vegetation and soil variables. All surfaces were processed with R v.3.3.2 (R Core Team, 2016) and resampled to a resolution of 5 m for landscape genetics analysis.

### 2.4 Landscape genetics analyses

We followed the optimization framework developed by Peterman et al. (2014) to determine the resistance values of our surfaces. Briefly, this approach uses monomolecular and Ricker functions (Bolker, 2008) to transform continuous resistance surfaces; it relies on a genetic algorithm (GA; Scrucca, 2013) that adaptively explores the parameter space, seeking to maximize the relationship between pairwise landscape distances (least‐cost or resistance) and pairwise genetic distances, while making no a priori assumptions about their relationships. The monomolecular [y = *r* (1‐exp^‐^ *^bx^*)] and Ricker [y = *r* exp^‐^ *^bx^*] are two exponential‐based functions used for ecological modeling, differing in the curve shape of the relationship they are modeling. This curve shape is mainly determined by shape (*x*) and magnitude (*b*) parameters, which produce a saturating exponential (growth or decay) curve for the monomolecular function, and a hump‐shaped curve (skewed to right or left) for the Ricker function (Bolker, 2008). During the optimization process, the genetic algorithm searches all possible combinations of these parameters for transforming resistance surfaces, denoted by “*r*” in the monomolecular and Ricker equations (Peterman, 2018; Peterman et al., 2014).

The optimization framework was performed with ResistanceGA in R (Peterman, 2018; https://github.com/wpeter man/ResistanceGA) following two steps: First, all surfaces were independently optimized from pairwise resistance distances estimated with gdistance in R (Van Etten, 2017), with the *commuteDistance* function, by exploring resistance values up to 2,500. Previous studies using this optimization approach (e.g., Peterman et al., 2014, Ruiz‐Lopez et al., 2016, Khimoun et al., 2017) have measured resistance distance using circuitscape (McRae, 2006); however, it is known that *commuteDistance* is functionally equivalent to circuitscape, with the advantage that it can be run in parallel (Kivimäki, Shimbo, & Saerens, 2014; Peterman, 2018). All these processes were performed using an eight‐neighbor connection scheme for assessing connectivity. We conducted three independent optimization runs for each surface to confirm convergence and parameter estimates (see the Appendix S1 for the R optimization scripts).

We used AIC as our objective function during optimization, which was determined from linear mixed‐effects models (lmem). The lmem were fitted by the maximum‐likelihood population effects (MLPE) parameterization, to account for the nonindependence of values within pairwise distance matrices (Clarke, Rothery, & Raybould, 2002; Van‐Strien, Keller, & Holderegger, 2012). The dependent and predictor variables were pairwise genetic distance (*D*_PS_) and pairwise scaled and centered resistance distance, respectively. MLPE parameterization was done with lme4 (Bates, Maechler, Bolker, & Walker, 2014) in R, and support of the optimized resistance surfaces was assessed using the AICc (Akaike's information criterion corrected for small/finite sample size; Akaike, 1974). To evaluate the robustness of our model selection and optimization given different combinations of samples, we performed a bootstrap resampling of the data (Peterman et al., 2014; Ruiz‐Lopez et al., 2016). Next, to control for potential bias in our results, 75% of the samples were randomly selected without replacement and each surface was then fit to the subset of individuals; the average rank, average model weight, and the percentage that a surface was selected as the best (top ranked) model following 10 000 iterations were estimated. Before the second optimization step, we did a Spearman coefficient correlation test (rho =ρ) with R between the surfaces that showed a greater selection percentage than distance alone, and selected, based on Cohen (1992), the surfaces that showed a small to medium correlation (ρ < 0.29), in order to avoid correlated variables in the multisurface model. Finally, we performed a multisurface optimization for selected surfaces to generate composite surfaces. Bootstrap model selection was performed again (75% of samples and 10,000 iterations) to obtain the average rank, average model weight, and the top ranked model of individual (i.e., univariate) and composite surfaces.

### 2.5 Radiotracking

We selected 17 individuals from three different webs along one transect (Figure 1), where trapping success was highest; each individual was equipped with a TXB‐003G radiotransmitter (Telenax, Mexico) attached to the suprascapular area with a drop of instant‐dry glue. The radiotransmitters weighed ca. 0.6 g, representing 1.3%–2% of the animals’ body weight, which is below the 5% maximum proposed by White and Garrot (1990). Radiolocations were taken one day after the individuals had been released (Springer, 2003), using a handheld three‐element Yagi directional antenna and an RX‐TLNX receiver (Telenax), between 21:00 and 01:30 hr, with intervals of 30 min and never recording the same individual consecutively, assuring data independence (Kernohan et al., 2001). Radiolocations were taken every night until reaching 10 per individual; georeferenced data, time, associated vegetation, and if the individual was actually observed were recorded for each one.

We used a probability‐based statistical estimator, the Kernel estimator, to calculate home range size (kernel estimating functions) with adehabitatHR (Calenge, 2015) in R v.3.3.0, using the smoothing parameters h_1CSV_ and h_ref_; area estimation did not differ between them thus we report the latter because it exhibited a better resolution for the isopleths that delimit regions with different probability within home ranges. This method estimates the intensity of area use as a two‐dimensional relative frequency distribution of an animal's location over time (Worton, 1989), avoiding biases due to its low sensitivity for extreme data (Rodgers & Carr, 1998) that other methods have (e.g., the minimum convex polygon). We tested for potential differences between female and male home range sizes using nonparametric Mann–Whitney U tests and estimated their overlap percentage, with R. We also estimated the Euclidian distance between radiolocations tracked consecutively for each individual, and based on the time of each radiolocation, we evaluated activity peaks considering the maximum distances the individuals moved during each radiotracking night. Finally, we recorded an indirect estimate of habitat use as the presence (percentage) of a vegetation type per radiolocation; differences between sexes were analyzed with Mann–Whitney U tests.

## 3 RESULTS

### 3.1 Population genetics

A total of 76 individuals were captured over the study area, 18 individuals in transect 1 (T1), 37 in T2, and 21 in T3. Eight microsatellite loci were polymorphic across all samples (Supporting Information Table S1 in the Appendix S1), whereas locus DS109 was monomorphic and was excluded from the analyses. Five loci (Ds1, Ds3, Ds19, Ds46, and DS98) deviated significantly from HWE after Bonferroni correction, while there was no evidence of LD (Bonferroni corrected *p* < 0.05); these same loci showed evidence of null alleles but not stuttering errors were detected. Because *D. merriami*‘s social structure encompasses groups of individuals with different degrees of relatedness (see relatedness results below), some evidence of deviation from HWE or null alleles is expected (i.e., not resulting from a systematic nonamplification of alleles; Bergl & Vigilant, 2007; Mapelli, Mora, Mirol, & Kittlein, 2012). Hence, all eight loci were included in the following analyses. Notably, we amplified a locus (Ds46) previously reported as unsuccessful in *D. merriami*, and we found that Ds19 is not a X‐linked locus in this species (Davis et al., 2000).

Genetic diversity results showed that the number of alleles per locus ranged from 8 to 30 (mean=15.1) and the number of effective alleles from 3.2 to 17.7 (mean=8.16). The mean observed heterozygosity and the unbiased expected heterozygosity across loci were 0.65 and 0.84, respectively; overall *F*_IS_ was 0.22 (Supporting Information Table S2 in the Appendix S1). Regarding relatedness, we detected 27 first‐order relationships (full siblings and parent–offspring), 11 occurred in different web/transect, six occurred in different webs along the same transect, and 10 occurred in the same web/transect. After removing one individual from each dyad, we obtained a dataset with 59 individuals (hereafter, unrelated dataset), used to test the effect of close relatives in population genetic structure.

Results about population genetic structure using structure and DAPC with both datasets were equivocal. Five clusters were detected by structure for the full dataset (*n* = 76), with both statistics (mean lnP(*K*) = −2,628.435 and ∆*K* = 12.331) (Supporting Information Figure S2a in the Appendix S1). However, the genetic clusters showed pairwise *F*_ST_ values of 0.027 to 0.239 and had no congruent geographic spatial pattern (Supporting Information Figure S2b in the Appendix S1). On the other hand, the curve of Bayesian information criterion (BIC) values for the DAPC results suggested the presence of *K* = 1 to *K* = 3 genetic clusters; notably, BIC values decreased rapidly, reaching their lowest value at *K* = 2 before rising again (Supporting Information Figure S3 in the Appendix S1). Because multiple possible *K* values have been described as a characteristic scenario for continuously distributed species using this method (Jombart, 2008), we performed DAPC with each the *K* = 2 and *K* = 3 assigned clusters by retaining 20 PCs, which comprised 71.7% of the total variance (Supporting Information Figure S3a in the Appendix S1). In both cases, groups were discriminated but in which clusters are not spatially structured; also, *F*_ST_ values between clusters were low (0.04 for *K* = 2; 0.042– 0.059 for *K* = 3).

Regarding the unrelated dataset (*n* = 59), one genetic cluster was obtained with structure (*K* = 1) by the mean lnP(*K*) = −2,195.545, whereas 11 clusters were detected with ∆*K* (20.781) (Supporting Information Figure S2c). For the DAPC, we retained all 59 PCs for the first step in the function *find.clusters* (Supporting Information Figure S4a in the Appendix S1). Results with DAPC were the same as those obtained with the full dataset; hence, we also applied DAPC to *K* = 2 and *K* = 3 (retaining 12 PCs and comprising 56.5% of the total variance), obtaining differentiation between groups but with clusters overlapping spatially (Supporting Information Figure S4b,c in the Appendix S1); *F*_ST_ values were again markedly low (0.043 for *K* = 2, 0.05–0.073 for *K* = 3).

Differences between structure and DAPC for detecting genetic structure in our study system can be explained by their assumptions. DAPC requires predefined groups, making this decision crucial for downstream interpretation of genetic data. In this context, clusters can be visualized as tools to summarize and understand the data, but recognizing that complex systems are not always subject to this clear‐cut representation (Jombart & Collins, 2015). As mentioned above, obtaining multiple values of *K* using the K‐means algorithm has been related to continuously distributed species, but only on a wide geographic extent (Guerrero et al., 2018), hence performing better for island‐based models than for continuous models (Jombart & Collins, 2015). Moreover, this algorithm uses a simple measure of group differentiation and is likely to fail to identify the correct number of clusters in complex population models (Jombart et al., 2010).

On the other hand, discrepancies between the structureresults for the full and the unrelated datasets, based on ∆*K*, agree with a family‐induced population structure (Anderson & Dunham, 2008); in addition, ∆*K* does not perform adequately when *K* = 1 (Evanno et al., 2005). Consequently, we consider results support one genetic cluster (no structuring) based on the above analytical issues, the low *F*_ST_ values between suggested clusters, the fine‐spatial scale of the study area, and importantly, the social structure of *D. merriami*.

### 3.2 Landscape genetics and connectivity

**Figure 2:**
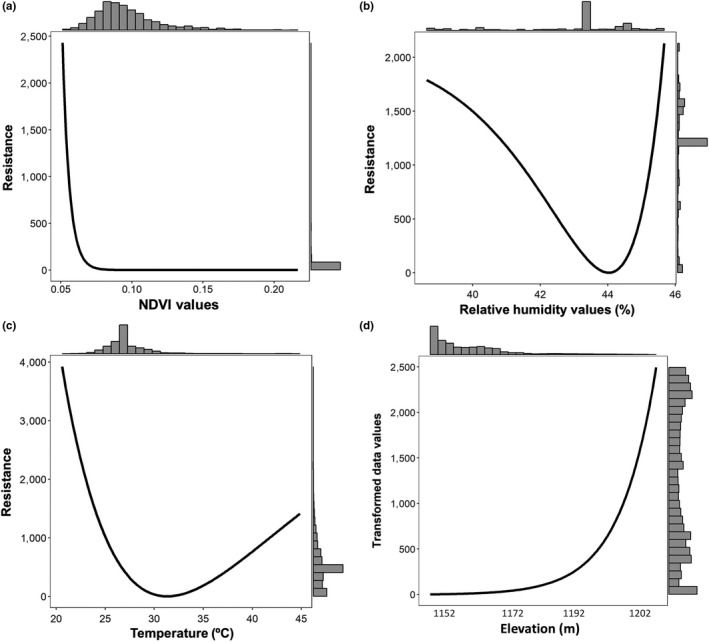
Single surface optimization response curves for (a) NDVI, (b) humidity, (c) temperature, and d) elevation, on genetic distance (*D_PS_*) for *Dipodomys merriami* individuals from the Mapimí Biosphere Reserve, Mexico. (a) NDVI was the best‐supported model (54.8% of the times based on 10 000 bootstrap replicates; Table 1), with an inverse monomolecular function, assigning high resistance to areas of the landscape with predominantly bare soil (<0.1), with a fast decrease in resistance where vegetation is present (>0.1), followed by (b) humidity (inverse–reverse Ricker), (c) temperature (inverse Ricker), and (d) elevation (inverse–reverse monomolecular).

Genetic distance (*D*_PS_) between individuals for LG analyses was estimated based on the unrelated dataset (Supporting Information Figure S5 in the Appendix S1). The Mantel test showed a nonsignificant negative correlation between the genetic and Euclidean distances (*r* = −0.022; *p* > 0.3), while the Mantel correlogram exhibited only one small positive and significant value at 1,700 m (*r* = 0.073; *p* < 0.05) (Supporting Information Figure S6 in the Appendix S1). Optimization and model selection results showed that the vegetation cover and soil resistance surface obtained with the NDVI were the best‐supported model (54.8% of the times based on 10,000 bootstrap replicates; Table 1), with an inverse monomolecular function (Figure 2a). Furthermore, NDVI was a significant predictor of genetic distance on the generalized linear mixed‐effects model (Supporting Information Table S3 in the Appendix S1). The NDVI optimized surface assigned high resistance to areas of the landscape with predominantly bare soil (<0.1), with a fast decrease in resistance where vegetation is present (> 0.1) (Figure 2a).

**Table 1:** Model selection results for the generalized linear mixed‐effects models optimized on genetic distance (*D*_PS_) for *Dipodomys merriami*

Surface	k	Equation	AIC	Average weight	Average rank	Top model (%)
NDVI	4	Inverse monomolecular	−3,320.1473	0.317	2.041	54.79
Humidity	4	Inverse–reverse Ricker	−3,319.2125	0.218	2.307	30.09
Elevation	4	Inverse–reverse monomolecular	−3,317.8299	0.145	3.413	4.19
Temperature	4	Inverse Ricker	−3,317.4376	0.126	3.922	4.91
Distance	2	NA	−3,316.2947	0.098	4.585	3.41
Feature^a^	3	NA	−3,316.3356	0.095	4.732	2.61
Null	1	NA	−3,318.267	NA	NA	NA

Note

*k* indicates the number of parameters in the transformation of continuous surfaces plus the intercept, or number of categories in binary surface plus the intercept

a Feature corresponds to the binary (presence/absence) classification of vegetation.

The following best‐supported functional form was humidity (inverse–reverse Ricker), with different functional forms for temperature (inverse Ricker) and elevation (inverse–reverse monomolecular), in which resistance values were lowest around 44% humidity, 30ºC temperature, and 1,152 m (Figure 2b,c,d). Additionally, humidity, elevation, and temperature resistance surfaces explained 30%, 4.2%, and 4.9%, respectively, of the variation in the pairwise genetic data than distance alone (Table 1), while vegetation and soil surface based on a binary classification (Feature surface) had a poor performance. Thus, we tested for correlations between NDVI, humidity, elevation, and temperature layers, where three pairwise comparisons showed evidence of an intermediate correlation (Supporting Information Table S4 in the Appendix S1). Accordingly, we constructed composite surfaces with these three comparisons, and also one including all four layers. Results of the bootstrap model selection showed that NDVI was the best‐supported model (54.9%), followed by humidity (19.7%) and NDVI+humidity (10.7%) (Table 2; the contribution of each variable to each multisurface model is shown in Table 3). The surface including all four layers (Combination 4) had no support and performed poorly in the generalized linear mixed‐effects model (Supporting Information Table S5 in the Appendix S1).

**Table 2:** Model selection results for both individual and composite surfaces for *Dipodomys merriami*

Surface	k	AIC	Average weight	Average rank	Top model (%)	Variables
NDVI	4	−3,320.147	0.255	2.588	54.9	NDVI
Humidity	4	−3,319.213	0.155	2.818	19.72	Humidity
Combination 1	5	−3,319.235	0.154	3.151	10.69	NDVI, humidity
Combination 3	5	−3,317.837	0.074	5.771	5.3	Temperature, elevation
Elevation	4	−3,317.83	0.107	4.420	4.4	Elevation
Combination 2	5	−3,317.432	0.092	5.386	2.56	NDVI, temperature
Temperature	4	−3,317.438	0.093	4.824	2.43	Temperature
Combination 4	9	−3,316.294	0.070	7.043	0	NDVI, humidity, temperature, elevation
Null	1	−3,318.267	NA	NA	NA	NA

Note

*k* indicates the number of parameters in the transformation of continuous surfaces plus the intercept, or number of categories in binary surface plus the intercept.

**Table 3:** Mean contribution of each variable to the corresponding multisurface model evaluated

Model	Variables	Mean contribution to model (%)
Combination 1	NDVI	1.4
	Humidity	98.6
Combination 3	Temperature	50.0
	Elevation	50.0
Combination 2	NDVI	1.0
	Temperature	99.0
Combination 4	NDVI	25.0
	Humidity	25.0
	Temperature	25.0
	Elevation	25.0

Note

Models are ranked in accordance with the model selection results in Table 2.

### 3.3 Home range

**Figure 3:**
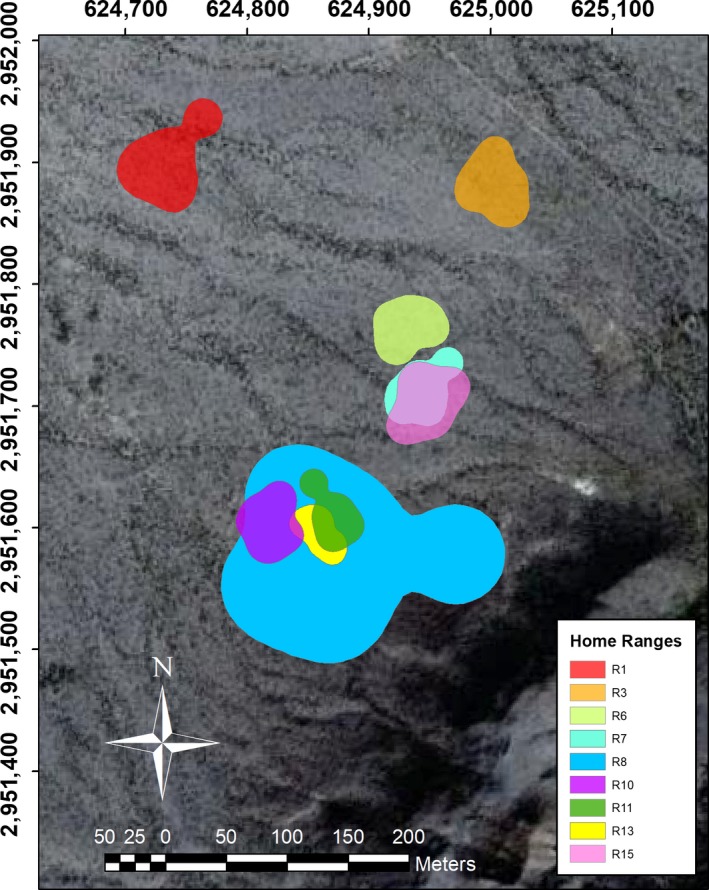
Home ranges of nine *Dipodomys merriami* individuals on the Mapimí Biosphere Reserve, Mexico, obtained from 91 individual radiolocations (telemetry). Radio numbers R13 and R15 are females, R6 a juvenile, and the rest are males. Six direct movement observations were obtained: once the female R13 and male R11, and twice the juvenile R6 and male R3

Of the 17 individuals with radiotransmitter, we lost radio signal for eight before obtaining enough data for analyses; hence, we report the results for nine individuals (two females, six males, one juvenile, sex undetermined) (Table 4). We obtained 91 independent radiolocations, and six individuals were directly observed while moving. The majority of the radiolocations used to calculate home range size per individual are within the estimated area, which shows these are regular activity zones. The home range size estimated was on average 0.6294±0.264 ha, 0.6957±0.3770 ha for males and 0.2453 ha for females (Figure 3, Table 4), with significant differences between sexes (U = 45, *p* = 0.003). The largest home range was a male's (R8), completely overlapping with that of another male (R11) and a female (R13). A 77.4% home range overlap was observed between a male and a female (radios R7 and R15), while among R8, R10, R11, and R13 varied from 10.3% to 100% (Figure 3, Table 4). Only two of the nine individuals showed a second‐order relationship (half‐siblings), the R7 male and the R15 female.

**Table 4:** Home range size (HR) for nine *Dipodomys merriami* individuals from the Mapimí Biosphere Reserve, Mexico

Radio	Sex	Locations	HR (ha)
R1	M	12	0.441
R3	M	10	0.328
R6	‐	10	0.270
R7	M	10	0.277
R8	M	10	2.652
R10	M	10	0.271
R11	M	10	0.204
R13	F	10	0.150
R15	F	10	0.341
Total (mean ±SE)			**0.6294 ± 0.264**
*M* (mean ±SE)			0.6957 ± 0.377
H (mean ±SE)			0.2453 ± 0.0

Note

Radio number, sex (male: M; female: F), number of radiolocations, and estimated area (ha ±standard error) are indicated

**Figure 4:**
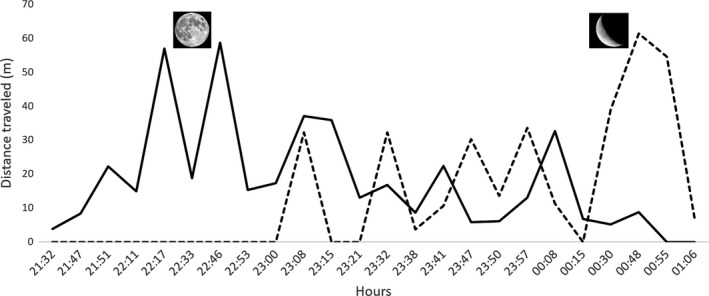
Activity cycles of nine *Dipodomys merriami* individuals on the Mapimí Biosphere Reserve, Mexico, obtained with radiolocations (telemetry), were recorded during 8 days, one from 21:30 to 00:55 (continuous line) during the full moon, and the other from 23:00 to 01:10 (dashed line) during the waning gibbous phase. The maximum distance travelled by an individual during each activity cycle was 56.8–58.6 m (at 22:17 and 22:46 hr) and 61.2 m (00:48), first and second cycles, respectively

Two activity cycles were recorded for the nine *D. merriami* individuals with radiolocations, during 8 days, one from 21:30 to 00:55, and the other from 23:00 to 01:10 (Figure 4). Three peaks were observed, that is the maximum distance travelled between two locations by an individual during each activity cycle, two by males R8 and R10 at 22:17 and 22:46 hr (56.8 m and 58.6 m, respectively) and the other of 61.2 m, by male R8 at 00:48. Interestingly, the first two peaks coincide with a full moon (first five sampling days), while the third was with decreasing illumination during the waning gibbous phase. Trapping sites had predominantly sandy and gravel soils, where the representative vegetation included six plant species: *Euphorbia antisyphilitica, Fouquieria splendens, Jatropha dioica, Larrea tridentata, Opuntia sp*., and *Prosopis glandulosa*. The highest percentage of individual radiolocations was on *L. tridentata* (57%), *Opuntia sp.* (40%), *J. dioica* (18%), and *P. glandulosa* (6%), with no significant differences between sexes.

## 4 DISCUSSION

### 4.1 Genetic diversity and microgeographic family‐induced structure

The genetic diversity patterns shown by *Dipodomys merriami* at the microgeographic area of the study are likely associated with the ecological complexity and social structure characterizing this desert‐dwelling species. Our results show moderate to high levels of genetic diversity when compared with other *Dipodomys* species (Busch, Waser, & DeWoody, 2007; Cosentino et al., 2015; Davis et al., 2000; Waser, Busch, McCormick, & DeWoody, 2006), with significant heterozygosity deficit estimates and a null genetic structure. Considering that trapping was conducted during the breeding season, our results may reflect movements of individuals across their home range area (interpopulation dispersal), generating a nonequilibrium pattern evidenced by heterozygote deficiency, a phenomenon observed for other *Dipodomys* species like the banner‐tailed kangaroo rat *D. spectabilis* (Busch et al., 2007). In this context, *D. merriami* exhibits a mean distance dispersal of around 60 m, while it has been suggested it can move longer distances during the breeding season with no distinction between sexes (Behrends et al., 1986; Zeng & Brown, 1987). Our results are in agreement, considering that the longest dispersal between radiolocations we observed was 57–61 m, performed by males. On the other hand, significant home range size differences between sexes were found (0.695 ha for males and 0.245 ha for females), despite being the breeding season; the latter can be related to less female movement compared with males due to gestation and offspring care (Murrieta‐Galindo & Cuatle‐García, 2016; Nader, 1978). Also, our findings show that females overlap home ranges with males but not between them; only two radiotracked individuals were related (half‐siblings), a male and a female that greatly overlapped their home ranges (70%). We acknowledge our low sample size and that results need be taken with caution; nonetheless, results are congruent with the behavior of *D. merriami* and support our prediction. Particularly, this species is considered a solitary and territorial rodent, characterized by adult male dispersal and phylopatric females that perform parental investment (Behrends et al., 1986; Randall, 1993).

Our results showed both no effect of geographic distance on genetic differentiation of *D. merriami* and no genetic structuring within the scale studied. Additionally, *D. merriami* exhibits frequent burrow shifts to avoid predators (Behrends et al., 1986), which has been related to a long‐life span (up to four years; Zeng & Brown, 1987), and consequently, a long‐term stability of populations that can result in a social phenomenon of tolerance by individual familiarity and mate selection (Behrends et al., 1986), leading to mating events between close neighbors (Randall, 1993). Hence, our overall *F*_IS_ results and lack of population genetic structure agree with a family‐induced pattern driven by first‐order‐related individuals, a biologically meaningful aspect of *D. merriami* described by ecological and behavioral data (Randall, 1993; Zeng & Brown, 1987), and now supported by the genetic component. Our study evidences the implications that a family‐induced structure at a microgeographic scale can have on landscape genetic inferences, specifically for selecting the unit level (i.e., populations or individuals) and genetic differentiation metrics for analyses (Shirk et al., 2017). Indeed, a correct assessment of population structure should always be conducted keeping in mind the more biologically relevant patterns (Anderson & Dunham, 2008; Bergl & Vigilant, 2007; Rodríguez‐Ramilo & Wang, 2012; Ruiz‐Lopez et al., 2016).

### 4.2 Environmental features and genetic connectivity

Our findings that gene flow in *D. merriami* is best explained by the normalized difference vegetation index (NDVI) enabled us to suggest a link between the observed genetic pattern and the ecological processes underlying it (e.g., the species’ dispersal, foraging, physiology). Finding these mechanistic links in landscape genetics research is one of the goals of the optimization framework we implemented (Peterman, 2018). The simultaneous optimization of multiple resistance surfaces (RS) and our creating a vegetation cover and soil texture surface based on the NDVI allowed us to explain the variation in genetic data better than any other individual or composite surface, suggesting these features are biologically relevant for *D. merriami* (Peterman et al., 2014; Ruiz‐Lopez et al., 2016; Spear et al., 2010). Although it has been suggested that multiple RS should be used for better capturing the landscape's realism (Spear et al., 2010, 2015), results do vary; for instance, Ruiz‐Lopez et al. (2016) found that a composite surface comprised of fire density and the distance to the nearest village describes the variation in genetic data of the red colobus monkey (*Procolobus gordonorum*), suggesting a strong influence of anthropogenic activities on the species movement. On the other hand, Khimoun et al. (2017) showed that individual optimization of land cover RS has a higher support compared to composite surfaces in the insular tropical bird Plumbeous warbler (*Setophaga plumbea*).

According to our expectations, we identified that vegetation cover is a key landscape feature for *D. merriami*, exhibiting that gene flow is strongly limited on open areas and facilitated where shrub cover with sandy and gravel soils is present. The role of vegetation as a facilitator of gene flow has been demonstrated for other rodent species occupying a variety of habitats with different levels of heterogeneity. For instance, Munshi‐South (2012) showed that gene flow in *Peromyscus leucopus* is determined by canopy cover in New York City, a generalist species inhabiting a highly urbanized landscape, while vegetation was strongly correlated with gene flow in *Mastomys natalensis*, a small and generalist rodent inhabiting the savanna of South Africa (Russo et al., 2016). Another example showed that forested areas in a tropical dry forest function as corridors for dispersal for the spiny pocket mice *Liomys pictus*, overcoming the potential limiting effect of roads across the landscape (Garrido‐Garduño et al., 2015); forests also facilitate gene flow in chipmunks (*Tamias striatus*) inhabiting fragmented landscapes like agroecosystems (Kierepka et al., 2016). Moreover, *D. merriami* activity peaks exhibit a behavior tightly associated with predator avoidance (Daly, Behrends, Wilson, & Jacobs, 1992; Soltz‐Herman & Valone, 2000), where movement is limited not only on open areas but also by lunar light (Daly et al., 1992; Fuentes‐Montemayor et al., 2009). Indeed, as our activity cycle results show, *D. merriami* is more active at crepuscular hours during full moon, while as the moonlight decreases its activity increases at midnight.

Interestingly, despite studies indicate that *D. merriami* feeds preferentially on seeds of *Prosopis glandulosa* and builds its burrows under this mesquite (Cox, De Alba‐Avila, Rice, & Cox, 1993; Reynolds, 1958), the dominant vegetation type on our study region is creosote (*Larrea tridentata*). Indeed, *D. merriami* exhibits a wider habitat use that includes preferentially *L. tridentata*, but also *Opuntia sp*., *J. dioica*, and *P. glandulosa*, highlighting the key role of this generalist species on desert ecosystem dynamics (Brown & Heske, 1990b; Murrieta‐Galindo & Cuatle‐García, 2016). Soil type, in this case sandy and gravel soils, is also a key factor associated with the plant species present, burrow construction, and individual movement within *D. merriami*'s home range.

Most landscape genetics inference studies have assumed either a positive or negative linear relationship between landscape features and cost surfaces (Garroway, Bowman, & Wilson, 2011; Koen, Bowman, & Walpole, 2012), including examples with rodent populations (Chiappero et al., 2016; Howell et al., 2017; Mora et al., 2017; Ortiz et al., 2017), despite that nonlinear responses are expected to be more common (Marrotte & Bowman, 2017; Spear et al., 2015). Here, we found nonlinear relationships relative to landscape resistance for both humidity and temperature, variables that influence genetic connectivity in different species like the northern quoll (*Dasyurus hallucatus*; Hohnen et al., 2016) and *Liomys pictus* (Garrido‐Garduño et al., 2015). Temperature has been explicitly proposed as determinant for genetic connectivity in climate sensitive species, for example, the American pika (*Ochotona princeps*), a heat intolerant, and cool microclimate restricted species for which an increase in temperature adversely affects gene flow (Castillo, Epps, Davis, & Cushman, 2014). Given the microgeographic scale of our study compared to the above mentioned, the nonlinear relationships we find may be associated with some aspects of *D. merriami*'s physiology, directly related to connectivity across the landscape. The thermoneutral zone (TNZ) refers to the temperature gradient where an organism's metabolism is minimized but also leads to higher rates of water loss, which ranges between 29º and 34ºC for this species (French, 1993). As a desert‐dwelling mammal, *D. merriami* has evolved certain physiological traits, for instance during its nocturnal active phase, it selects cooler TNZ temperatures (30.3–31.5ºC) for water conservation (Banta, 2003). Also, it is known that 40% humidity favors its movement on the surface (i.e., not across their burrows underground), whereas values outside this range may be detrimental (Frank, 1988; Reynolds, 1958; Walsberg, 2000). Indeed, our results show that resistance is lowest around 44% humidity and 30ºC, in agreement with *D. merriami*‘s TNZ and humidity ranges. Moreover, these variables follow an inverse–reverse Ricker and inverse Ricker functional forms, respectively, reflecting “optimal peaks” (i.e., physiological meaningful variables) that promote gene flow across the desert environment. At the same time, these nonlinear responses exhibit the limits imposed to kangaroo rats by the desert conditions, directly impacting its genetic connectivity. Such a fine‐dependence on local microclimate has been observed for the terrestrial woodland salamander (*Plethodon albagula*; Peterman et al., 2014).

### 4.3 Thematic resolution for detecting landscape genetic patterns

Scale has been recognized as a central question in ecology (Levin, 1992). In landscape genetics studies, particular attributes determine the strength and nature of observed pattern‐process relationships. Specifically, landscape scales (landscape extent and thematic resolution) are crucial and need be defined objectively (Cushman & Landguth, 2010; Khimoun et al., 2017; Spear et al., 2015; Wasserman, Cushman, Schwartz, & Wallin, 2010). Indeed, the pixel size and the thematic resolution of the native data should be considered when obtaining (from available sources) or creating (from field or empirical data) landscape resistance surfaces (RS), always keeping in mind the system being analyzed. From the two approaches used to represent vegetation in our study, the Landsat‐NDVI continuous RS was selected as the best model despite its low native resolution (30 m) when compared to Google Earth binary RS with high native resolution (approximately 1.3 m). Given the microgeographic study area and *D. merriami*'s small body size and short dispersal distances, we expected that a native finer resolution, namely pixel size coupled with a classification based on vegetation field data, would be better than a native coarser resolution, being biologically relevant and providing a more detailed structure of the landscape for adequately evaluating connectivity (Boyle et al., 2014; Sawyer, Epps, & Brashares, 2011; Zeller et al., 2011). However, thematic resolution, that is, how finely are the landscape variables represented, either as categorical or continuous surfaces, is the scale‐related attribute that determines the most the detection of landscape genetic patterns (Cushman & Landguth, 2010; Khimoun et al., 2017; Wasserman et al., 2010). Hence, caution has been suggested regarding the level of detail in surface classification, specifically when transforming surfaces from continuous to categorical. In fact, Cushman and Landguth (2010) showed, with a simulation study, that categorical maps do not represent adequately continuous processes in LG. Additionally, empirical examples with two species having markedly different ecological traits, the American marten (*Martes americana*; Wasserman et al., 2010) and the plumbeous warbler (Khimoun et al., 2017), showed that landscape definition based on alternative classification schemes may lead to erroneous detection of landscape effects on gene flow. Also, in a study with the arboreal and forest adapted Udzungwa red colobus monkey, authors represented current and historical forest cover as categorical surfaces, but argued that surprisingly neither were good predictors of genetic differentiation (Ruiz‐Lopez et al., 2016).

Our study and the above examples reflect the importance of scale in LG research, showing that categorical surfaces, in general, may fail to adequately represent the relationship between landscape and genetic data, as highlighted by Cushman and Landguth (2010). In addition, the two approaches we used to represent the same feature at different scale attributes partially allowed us to exhibit their effects at a microgeographic scale, where vegetation was better represented as continuously distributed, thus evidencing the need to characterize that feature according to its “more‐real” nature. Finally, there are recent advances aimed to make categorical data more ecologically relevant in LG research (Peterman, 2018), although they still need to be evaluated empirically. Our study is an empirical example of how the pixel size and thematic resolution need be considered, particularly for small body size species and at microgeographic areas, when developing RS that are relevant to the study system.

## 5 CONCLUSIONS

Evaluating the effects of landscape features (landscape matrix) on individual movement and gene flow in natural populations has been challenged by the need to avoid subjectivity when assigning resistance values and landscape scales (Khimoun et al., 2017; Richardson et al., 2016; Spear et al., 2010). Our study is novel in diverse aspects, where we present a landscape genetics study with a desert‐dwelling rodent species—an ecosystem rarely investigated under this genetics approach—using a nonsubjective optimization framework for resistance surfaces and an accurate definition of thematic resolution (Khimoun et al., 2017; Peterman, 2018; Peterman et al., 2014). Furthermore, we describe new information about the genetic variability, ecology, and behavior of the Merriam's kangaroo rat *Dipodomys merriami* at its southern distribution that adds to the limited studies mostly performed on the northern Chihuahuan Desert region, including the different approaches used to represent landscape and environmental features and their effects at a microgeographic scale; also, by the thematic resolution comparisons and how to best represent (categorical or continuous) surfaces. Moreover, our estimation of the species’ home range, habitat use, and activity based on telemetry and a probability‐based statistical kernel estimator, not only significantly contributed to the interpretation of *D. merriami*'s functional connectivity, but it is also one of the first studies using telemetry performed with rodents (Marines‐Macías, Colunga‐Salas, Verde‐Arregoitia, Naranjo, & León‐Paniagua, 2018). Finally, our study evidences both the importance of having a correct assessment of population structure based on biologically relevant patterns and, importantly, the implications that a family‐induced structure can have on landscape genetic inferences. Accordingly, we were able to derive individual and composite surfaces and adequately test their relationship with *D. merriami*'s interindividual genetic distances, showing that vegetation cover, soil texture, and climatic variables like humidity influence its functional connectivity. Based on our overall results, we describe patterns of the relationship between environmental features and some aspects of the behavior and physiology of *D. merriami*.

## CONFLICTS OF INTEREST

The authors declare that they have no conflicts of interest.

## AUTHOR CONTRIBUTIONS

A.F.M., M.A.L.B. and E.V.D. conceptualized and designed the research; also performed fieldwork with other collaborators. A.F.M. performed laboratory work. A.F.M. and M.A.L.B. conducted data analyses with contributions of R.J.D. and E.V.D. All authors contributed in the interpretation of results and writing of the paper.

## DATA ACCESSIBILITY

Microsatellites genotypes are available from the Dryad Digital Repository at doi:10.5061/dryad.dk73qp7

## References

[ece34762-bib-0001] Akaike, H. (1974). A new look at the statistical model identification. IEEE Transactions on Automatic Control, 19, 716–723. 10.1109/TAC.1974.1100705

[ece34762-bib-0002] Anderson, D. R. , Burnham, K. P. , White, G. C. , & Otis, D. L. (1983). Density estimation of small‐mammal populations using a trapping web and distance sampling methods. Ecology, 64, 674–680. 10.2307/1937188

[ece34762-bib-0003] Anderson, E. C. , & Dunham, K. K. (2008). The influence of family groups on inferences made with the program structure. Molecular Ecology Resources, 8, 1219–1229. 10.1111/j.1755-0998.2008.02355.x 21586009

[ece34762-bib-0004] Aragón, E. E. , Castillo, B. , & Garza, A. (2002). Roedores en la dieta de dos aves rapaces nocturnas (*Bubo virginianus* y *Tyto alba*) en el noreste de Durango, México. Acta Zoológica Mexicana (nueva serie), 86, 29–50.

[ece34762-bib-0005] Balkenhol, N. , Cushman, S. A. , Storfer, A. , & Waits, L. P. (2015). Introduction to Landscape Genetics – Concepts, Methods, Applications In Balkenhol N., Cushman S. A., Storfer A. T., & Waits L. P. (Eds.), Landscape Genetics: Concepts, Methods, Applications (pp. 1–7). Chichester, UK: John Wiley & Sons Ltd.

[ece34762-bib-0006] Banta, M. R. (2003). Merriam’s kangaroo rats (*Dipodomys merriami*) voluntarily select temperatures that conserve energy rather than water. Physiological and Biochemical Zoology, 76, 522–532. 13130431 10.1086/375437

[ece34762-bib-0007] Bates, D. M. , Maechler, M. , Bolker, B. M. , & Walker, S. (2014). Linear mixed‐effects models using Eigen and S4. R package version 1.1‐6. Retrieved from http://CRAN.R-project.org/package=gdistance=lme4

[ece34762-bib-0008] Behrends, P. , Daly, M. , & Wilson, M. I. (1986). Range use patterns and spatial relationships of Merriami’s kangaroo rats (*Dipodomys merriami*). Behaviour, 96, 187–209.

[ece34762-bib-0009] Bergl, R. A. , & Vigilant, L. (2007). Genetic analysis reveals population structure and recent migration within the highly fragmented range of the Cross River gorilla (*Gorilla gorilla diehli*). Molecular Ecology, 16, 501–516. 10.1111/j.1365-294X.2006.03159.x 17257109

[ece34762-bib-0010] Bolker, B. (2008). Ecological models and data in R. Princeton, NJ: Princeton University Press.

[ece34762-bib-0011] Boyle, S. A. , Kennedy, C. M. , Torres, J. , Colman, K. , Pérez‐Estigarribia, P. E. , & de la Sancha, N. U. (2014). High‐resolution satellite imagery is an important yet underutilized resource in conservation biology. PLoS ONE, 9, e86908 10.1371/journal.pone.0086908 24466287 PMC3900690

[ece34762-bib-0012] Brown, J. H. , & Heske, E. J. (1990a). Temporal changes in a Chihuahuan desert rodent community. Oikos, 59, 290–302.

[ece34762-bib-0013] Brown, J. H. , & Heske, E. J. (1990b). Control of a desert‐grassland transition by a keystone rodent guild. Science, 250, 1705–1707. 17734708 10.1126/science.250.4988.1705

[ece34762-bib-0014] Burt, W. H. (1943). Territoriality and home range concepts as applied to mammals. Journal of Mammalogy, 24, 346–352. 10.2307/1374834

[ece34762-bib-0015] Busch, J. D. , Waser, P. M. , & DeWoody, A. (2007). Recent demographic bottlenecks are not accompanied by a genetic signature in banner‐tailed kangaroo rats (*Dipodomys spectabilis*). Molecular Ecology, 16, 2450–2462. 10.1111/j.1365-294X.2007.03283.x 17561905

[ece34762-bib-0016] Calenge, C. (2015). Home range estimation in R: The adehabitatHR package. Retrieved from https://cran.r-project.org/web/packages/adehabitatHR

[ece34762-bib-0017] Castillo, J. A. , Epps, C. W. , Davis, A. R. , & Cushman, S. A. (2014). Landscape effects on gene flow for climate‐sensitive montane species, the American pika. Molecular Ecology, 23, 843–856. 24383818 10.1111/mec.12650

[ece34762-bib-0018] Chiappero, M. B. , Sommaro, L. V. , Priotto, J. W. , Wirnes, M. P. , Steinmann, A. R. , & Gardenal, C. N. (2016). Spatio‐temporal genetic structure of the rodent *Calomys venustus* in linear, fragmented habitats. Journal of Mammalogy, 97, 424–435.

[ece34762-bib-0019] Clarke, R. T. , Rothery, P. , & Raybould, A. F. (2002). Confidence limits for regression relationships between distance matrices: Estimating gene flow with distance. Journal of Agricultural, Biological and Environmental Statistics, 7, 361–372. 10.1198/108571102320

[ece34762-bib-0020] Cohen, J. (1992). A power primer. Phycological. Psychological Bulletin, 112, 155–159. 10.1037/0033-2909.112.1.155 19565683

[ece34762-bib-0021] Conanp . (2006). *Programa de conservación y manejo Reserva de la Biosfera Mapimí, México. Comisión Nacional de Áreas Naturales Protegidas*. Secretaría del Medio Ambiente y Recursos Naturales, México.

[ece34762-bib-0022] Cosentino, B. J. , Schooley, R. L. , Bestelmeyer, B. T. , McCarthy, A. J. , & Sierzega, K. (2015). Rapid genetic restoration of a keystone species exhibiting delayed demographic response. Molecular Ecology, 24, 6120–6133. 10.1111/mec.13469 26577599

[ece34762-bib-0023] Cox, J. R. , De Alba‐Avila, A. , Rice, R. W. , & Cox, J. N. (1993). Biological and physical factors influencing *Acacia constricta* and *Prosopis velutina* establishment in the Sonoran Desert. Journal of Range Management, 46, 43–48. 10.2307/4002446

[ece34762-bib-0024] Creech, T. G. , Epps, C. W. , Monello, R. J. , & Wehausen, J. D. (2014a). Using network theory to prioritize management in a desert bighorn sheep metapopulation. Landscape Ecology, 29, 605–619.

[ece34762-bib-0025] Creech, T. G. , Epps, C. W. , Monello, R. J. , & Wehausen, J. D. (2014b). Predicting diet quality and genetic diversity of a desert‐adapted ungulate with NDVI. Journal of Arid Environments, 127, 160–170.

[ece34762-bib-0026] Cushman, S. A. , & Landguth, E. L. (2010). Scale dependent inference in landscape genetics. Landscape Ecology, 25, 967–979. 10.1007/s10980-010-9467-0 20618896

[ece34762-bib-0027] Cushman, S. A. , Shirk, A. J. , & Landguth, E. L. (2013). Landscape genetics and limiting factors. Conservation Genetics, 14, 263–274. 10.1007/s10592-012-0396-0

[ece34762-bib-0028] Daly, M. , Behrends, P. R. , Wilson, M. I. , & Jacobs, L. F. (1992). Behavioral modulation of predation risk: Moonlight avoidance and crepuscular compensation in a nocturnal desert rodent *Dipodomys merriami* . Animal Behaviour, 44, 1–9.

[ece34762-bib-0029] Davis, C. , Keane, B. , Swanson, B. , Loew, S. , Waser, P. M. , Strobeck, C. , & Fleischer, R. C. (2000). Characterization of microsatellite loci in bannertailed and giant kangaroo rats, *Dipodomys spectabilis* and *Dipodomys ingens* . Molecular Ecology, 9, 642–644. 10.1046/j.1365-294x.2000.00882-8.x 10792713

[ece34762-bib-0030] Demšar, U. , Buchin, K. , Cagnacci, F. , Safi, K. , Speckmann, B. , Van de Weghe, N. , … Weibel, R. (2015). Analysis and visualization of movement: An interdisciplinary review. Movement Ecology, 3, 5. 25874114 10.1186/s40462-015-0032-y PMC4395897

[ece34762-bib-0031] Dyer, R. J. (2014). gstudio. An R Package for the Spatial Analysis of Population Genetic Data, (https://github.com/dyerlab/gstudio).

[ece34762-bib-0032] Dyer, R. J. (2015). Is there such a thing as landscape genetics? Molecular Ecology, 24, 3518–3528. 10.1111/mec.13249 26011166

[ece34762-bib-0033] Earl, D. A. , & vonHold, B. M. (2012). STRUCTURE HARVESTER: A website and program for visualizing STRUCTURE output and implementing the Evanno method. Conservation Genetics Resources, 4, 359–361. 10.1007/s12686-011-9548-7

[ece34762-bib-0034] Evanno, G. , Regnaut, S. , & Goudet, J. (2005). Detecting the number of clusters of individuals using the software STRUCTURE: A simulation study. Molecular Ecology, 14, 2611–2620. 10.1111/j.1365-294X.2005.02553.x 15969739

[ece34762-bib-0035] Frank, C. L. (1988). The influence of moisture content on seed selection by kangaroo rats. Journal of Mammalogy, 69, 353–357. 10.2307/1381385

[ece34762-bib-0036] French, A. R. (1993). Physiological ecology of the Heteromyidae: economics of energy and water utilization In Genoways H. H., & Brown J. H. (Eds.), Biology of the Heteromyidae. Special Publications of American Society of Mammalogists, 10, 509–538.

[ece34762-bib-0037] Fuentes‐Montemayor, E. , Cuarón, A. D. , Vázquez‐Domínguez, E. , Benítez‐Malvido, J. , Valenzuela, D. , & Andresen, E. (2009). Living on the edge: Roads and edge effects on small mammal populations. Journal of Animal Ecology, 78, 857–865. 10.1111/j.1365-2656.2009.01551.x 19426252

[ece34762-bib-0038] Gardner‐Santana, L. C. , Norris, D. E. , Fornadel, C. M. , Hinson, E. R. , Klein, S. L. , & Glass, G. E. (2009). Commensal ecology, urban landscapes, and their influence on the genetic characteristics of city‐dwelling Norway rats (*Rattus norvegicus*). Molecular Ecology, 18, 2766–2778. 19457177 10.1111/j.1365-294X.2009.04232.x PMC4118303

[ece34762-bib-0039] Garrido‐Garduño, T. , Téllez‐Valdés, O. , Manel, S. , & Vázquez‐Domínguez, E. (2015). Role of habitat heterogeneity and landscape connectivity in shaping gene flow and spatial population structure of a dominant rodent species in a tropical dry forest. Journal of Zoology, 298(4), 293–302. 10.1111/jzo.12307.

[ece34762-bib-0040] Garroway, C. J. , Bowman, J. , & Wilson, P. J. (2011). Using a genetic network to parameterize a landscape resistance surface for fishers, *Martes pennanti* . Molecular Ecology, 20, 3978–3988. 10.1111/j.1365-294X.2011.05243.x 21883589

[ece34762-bib-0041] Goslee, S. C. , & Urban, D. L. (2007). The ecodist package for dissimilarity‐based analysis of ecological data. Journal of Statistical Software, 22, 1–19.

[ece34762-bib-0042] Goudet, J. (2015). Package “hierfstat”. R package version 0.04‐22. Retrieved from http://www.r-project.org,http://github.com/jgx65/hierfstat

[ece34762-bib-0043] Grünberger, O. (2004). Características esenciales de la Reserva de la Biosfera In Grünberger O., Reyes‐Gómez V. M., & Janeau J. L. (Eds.), Las playas del desierto chihuahuense (parte mexicana): Influencia de las sales en ambiente árido y semiárido (pp. 41–55). Xalapa, Mexico: IRD‐INECOL.

[ece34762-bib-0044] Guerrero, J. , Byrne, A. W. , Lavery, J. , Presho, E. , Kelly, G. , Coursier, E. A. , … Allen, A. R. (2018). The population and landscape genetics of the European badger (*Meles meles*) in Ireland. Ecology and Evolution, 8, 10233–10246. 30397461 10.1002/ece3.4498 PMC6206220

[ece34762-bib-0045] Hernández, L. , Romero, A. G. , Laundré, J. W. , Lightfoot, D. , Aragón, E. , & Portillo, J. L. (2005). Changes in rodent community structure in the Chihuahuan Desert Mexico: Comparisons between two habitats. Journal of Arid Environments, 60, 239–257.

[ece34762-bib-0046] Hijmans, R. J. , Cameron, S. E. , Parra, J. L. , Jones, P. G. , & Jarvis, A. (2005). Very high resolution interpolated climate surfaces for global land areas. International Journal of Climatology, 25, 1965–1978. 10.1002/joc.1276

[ece34762-bib-0047] Hohnen, R. , Tuft, K. D. , Legge, S. , Hillyer, M. , Spencer, P. B. , Radford, I. J. , … Burridge, C. P. (2016). Rainfall and topography predict gene flow among populations of the declining northern quoll (*Dasyurus hallucatus*). Conservation Genetics, 17, 1213–1228. 10.1007/s10592-016-0856-z

[ece34762-bib-0048] Holdaway, M. R. (1996). Spatial modeling and interpolation of monthly temperature using kriging. Climate Research, 6, 215–225. 10.3354/cr006215

[ece34762-bib-0049] Howell, P. E. , Delgado, M. L. , & Scribner, K. T. (2017). Landscape genetic analysis of co‐distributed white‐footed mice (*Peromyscus leucopus*) and prairie deer mice (*Peromyscus maniculatus bairdii*) in an agroecosystem. Journal of Mammalogy, 98, 793–803. 10.1093/jmammal/gyx042

[ece34762-bib-0050] Jaquiéry, J. , Broquet, T. , Hirzel, A. H. , Yearsley, J. , & Perrin, N. (2011). Inferring landscape effects on dispersal from genetic distances: How far can we go? Molecular Ecology, 20, 692–705. 10.1111/j.1365-294X.2010.04966.x 21175906

[ece34762-bib-0051] Jombart, T. (2008). adegenet: a R package for the multivariate analysis of genetic markers. Bioinformatics, 24(11), 1403–1405. 10.1093/bioinformatics/btn129 18397895

[ece34762-bib-0052] Jombart, T. , & Collins, C. (2015). A tutorial for Discriminant Analysis of Principal Components (DAPC) using adegent 2.0.0. Retrieved from http://adegenet.r-forge.r-project.org/files/tutorial-dapc.pdf

[ece34762-bib-0053] Jombart, T. , Devillard, S. , & Balloux, F. (2010). Discriminant analysis of principal components: A new method for the analysis of genetically structured populations. BMC Genetics, 11, 94. 20950446 10.1186/1471-2156-11-94 PMC2973851

[ece34762-bib-0054] Jombart, T. , Kamvar, Z. N. , Lustrik, R. , Collins, C. , Beugin, M. P. , Knaus, B. , … Calboli, F. (2016). Exploratory Analysis of Genetic and Genomic Data. R Package Version, 2, 1. Retrieved from http://adegenet.r-forge.r-project.org/

[ece34762-bib-0055] Kalinowski, S. T. , Wagner, A. , & Taper, M. L. (2006). ML‐RELATE. A computer program for maximum likelihood estimation of relatedness and relationship. Molecular Ecology Notes, 6, 576–579.

[ece34762-bib-0056] Kernohan, B. J. , Gitzen, R. A. , & Millspaugh, J. J. (2001). Analysis of animal space use and movements In Millspaugh J. J., & Marzluff J. M. (Eds.), Radio tracking and animal populations (pp. 125–166). San Diego, CA: Academic Press.

[ece34762-bib-0057] Khimoun, A. , Peterman, W. , Eraud, C. , Faivre, B. , Navarro, N. , & Garnier, S. (2017). Landscape genetic analyses reveal fine‐scale effects of forest fragmentation in an insular tropical bird. Molecular Ecology, 26, 4906–4919. 10.1111/mec.14233 28727200

[ece34762-bib-0058] Kierepka, E. M. , Anderson, S. J. , Swihart, R. K. , & Rhodes, O. E. Jr (2016). Evaluating the influence of life‐history characteristics on genetic structure: A comparison of small mammals inhabiting complex agricultural landscapes. Ecology & Evolution, 6, 6376–6396. 10.1002/ece3.2269 27648250 PMC5016657

[ece34762-bib-0059] Kivimäki, I. , Shimbo, M. , & Saerens, M. (2014). Developments in the theory of randomized shortest paths with a comparison of graph node distances. Physica A: Statistical Mechanics and Its Applications, 393, 600–616. 10.1016/j.physa.2013.09.016

[ece34762-bib-0060] Koen, E. L. , Bowman, J. , & Walpole, A. A. (2012). The effect of cost surface parameterization on landscape resistance estimates. Molecular Ecology Resources, 12, 686–696. 10.1111/j.1755-0998.2012.03123.x 22353473

[ece34762-bib-0061] Levin, S. A. (1992). The problem of pattern and scale in ecology. Ecology, 73, 1943–1967.

[ece34762-bib-0062] Lonsinger, R. C. , Schweizer, R. M. , Pollinger, J. P. , Wayne, R. K. , & Roemer, G. W. (2015). Fine‐scale genetic structure of the ringtail (*Bassariscus astutus*) in a Sky Island mountain range. Journal of Mammalogy, 96, 257–268.

[ece34762-bib-0063] Ludwig, J. A. , Wilcox, B. P. , Breshears, D. D. , Tongway, D. J. , & Imeson, A. C. (2005). Vegetation patches and runoff‐erosion as interacting ecohydrological processes in semiarid landscapes. Ecology, 86, 288–297. 10.1890/03-0569

[ece34762-bib-0064] Manel, S. , Schwartz, M. K. , Luikart, G. , & Taberlet, P. (2003). Landscape genetics: Combining landscape ecology and population genetics. Trends in Ecology & Evolution, 4, 189–197. 10.1016/S0169-5347(03)00008-9

[ece34762-bib-0065] Mapelli, F. J. , Mora, M. S. , Mirol, P. M. , & Kittlein, M. J. (2012). Population structure and landscape genetics in the endangered subterranean rodent *Ctenomys porteousi* . Conservation Genetics, 13, 165–181. 10.1007/s10592-011-0273-2

[ece34762-bib-0066] Marines‐Macías, T. , Colunga‐Salas, P. , Verde‐Arregoitia, L. D. , Naranjo, E. J. , & León‐Paniagua, L. (2018). Space use by two arboreal rodent species in a Neotropical cloud forest. Journal of Natural History, 42, 1417–1431. 10.1080/00222933.2018.1459921

[ece34762-bib-0067] Marrotte, R. R. , & Bowman, J. (2017). The relationship between least‐cost and resistance distance. PLoS ONE, 12, e0174212 10.1371/journal.pone.0174212 28350863 PMC5369686

[ece34762-bib-0068] Martínez, E. , & Morello, J. (1977). El medio físico y las unidades fisonómico‐florístico del. Bolsón De Mapimí: Reserva De La Biosfera Mapimí. Publicaciones Del Instituto De Ecología, 3:63 p.

[ece34762-bib-0069] McRae, B. H. (2006). Isolation by resistance. Evolution, 60, 1551–1561. 10.1111/j.0014-3820.2006.tb00500.x 17017056

[ece34762-bib-0070] Montaña, C. (1988). Las formaciones vegetales In Montaña C. (Ed.), Estudio integrado de los recursos vegetación, suelo y agua en la Reserva de la Biosfera Mapimí: I. Ambiente natural y humano (pp. 167–197). Xalapa, México: Instituto de Ecología, A. C. México.

[ece34762-bib-0071] Montaña, C. , & Breimer, R. F. (1988). Major vegetation and environment units En Montaña C. (Ed.), Estudio integrado de los recursos vegetación, suelo y agua en la Reserva de la Biosfera Mapimí: I. Ambiente natural y humano (pp. 99–1114). Xalapa, México: Instituto de Ecología, A. C. México.

[ece34762-bib-0072] Mora, M. S. , Mapelli, F. J. , López, A. , Gómez‐Fernández, M. J. , Mirol, P. M. , & Kittlein, M. J. (2017). Landscape genetics in the subterranean rodent *Ctenomys “chasiquensis”* associated with highly disturbed habitats from the southeastern Pampas region, Argentina. Genetica, 145, 575–591. 10.1007/s10709-017-9983-9 28905157

[ece34762-bib-0073] Munshi‐South, J. (2012). Urban landscape genetics: Canopy cover predicts gene flow between white‐footed mouse (*Peromyscus leucopus*) populations in New York City. Molecular Ecology, 21, 1360–1378. 22320856 10.1111/j.1365-294X.2012.05476.x

[ece34762-bib-0074] Murray, J. V. , Goldizen, A. W. , O’Leary, R. A. , McAlpine, C. A. , Possingham, H. P. , & Choy, S. L. (2009). How useful is expert opinion for predicting the distribution of a species within and beyond the region of expertise? A case study using brush‐tailed rock‐wallabies *Petrogale penicillata* . Journal of Applied Ecology, 46, 842–851.

[ece34762-bib-0075] Murrieta‐Galindo, R. , & Cuatle‐García, L. M. (2016). Burrows of genus *Dipodomys* in two plant communities in the Mapimí Reserve Biosphere Durango, Mexico. RINDERESU, 1, 35–47.

[ece34762-bib-0076] Nader, I. A. (1978). Kangaroo rats: Intraspecific variation in Dipodomys spectabilis Merriam and Dipodomys deserti Stephens. Illinois Biological Monographs 49. Chicago, IL: University of Illinois Press.

[ece34762-bib-0077] Ortiz, N. , Polop, F. J. , Andreo, V. C. , Provensal, M. C. , Polop, J. J. , Gardenal, C. N. , & González‐Ittig, R. E. (2017). Genetic population structure of the long‐tailed pygmy rice rat (Rodentia, Cricetidae) at different geographic scales in the Argentinean Patagonia. Journal of Zoology, 301, 215–226. 10.1111/jzo.12410

[ece34762-bib-0078] Partridge, L. (1978). Habitat selection In Krebs J. R., & Davies N. B. (Eds.), Behavioral ecology and evolutionary approach (pp. 351–376).Oxford, UK: Blackwell.

[ece34762-bib-0079] Peterman, W. E. (2018). ResistanceGA: An R package for the optimization of resistance surfaces using genetic algorithms. Methods in Ecology & Evolution, 9, 1638–1647.

[ece34762-bib-0080] Peterman, W. E. , Connette, G. M. , Semlitsch, R. D. , & Eggert, L. S. (2014). Ecological resistance surfaces predict fine‐scale genetic differentiation in a terrestrial woodland salamander. Molecular Ecology, 23, 2402–2413. 10.1111/mec.12747 24712403

[ece34762-bib-0081] Portanier, E. , Larroque, J. , Garel, M. , Marchand, P. , Maillard, D. , Bourgoin, G. , & Devillard, S. (2018). Landscape genetics matches with behavioral ecology and brings new insight on the functional connectivity in Mediterranean mouflon. Landscape Ecology, 33, 1069–1085. 10.1007/s10980-018-0650-z

[ece34762-bib-0082] Powell, R. A. , & Mitchell, M. S. (2012). What is a home range? Journal of Mammalogy, 93, 948–958. 10.1644/11-MAMM-S-177.1

[ece34762-bib-0083] Pritchard, J. K. , Stephens, M. , & Donnelly, P. (2000). Inference of population structure using multilocus genotype data. Genetics, 155, 945–959. 10835412 10.1093/genetics/155.2.945 PMC1461096

[ece34762-bib-0084] R Core Team (2016). R: A language and environment for statistical computing. Vienna, Austria: R Foundation for Statistical Computing Retrieved from http://www.R-project.org/

[ece34762-bib-0085] Randall, J. A. (1993). Behavioural adaptations of desert rodents (Heteromyidae). Animal Behaviour, 45, 263–287. 10.1006/anbe.1993.1032

[ece34762-bib-0086] Reding, D. M. , Cushman, S. A. , Gosselink, T. E. , & Clark, W. R. (2013). Linking movement behavior and fine‐scale genetic structure to model landscape connectivity for bobcats (*Lynx rufus*). Landscape Ecology, 28, 471–486. 10.1007/s10980-012-9844-y

[ece34762-bib-0087] Reynolds, H. G. (1958). The ecology of the Merriam kangaroo rat (*Dipodomys merriami*) on the grazing lands of southern Arizona. Ecological Monographs, 28, 111–127.

[ece34762-bib-0088] Rice, W. R. (1989). Analyzing tables of statistical tests. Evolution, 43, 223–225. 10.1111/j.1558-5646.1989.tb04220.x 28568501

[ece34762-bib-0089] Richardson, J. L. , Brady, S. P. , Wang, I. J. , & Spear, S. F. (2016). Navigating the pitfalls and promise of landscape genetics. Molecular Ecology, 25, 849–863. 10.1111/mec.13527 26756865

[ece34762-bib-0090] Rodgers, A. R. , & Carr, A. P. (1998). HRE: The home range extension for ArcView, user’s manual. Centre for Northern Forest Ecosystem Research. Ontario Ministry of Natural Resources, 30.

[ece34762-bib-0091] Rodríguez‐Ramilo, S. T. , & Wang, J. (2012). The effect of close relatives on unsupervised Bayesian clustering algorithms in population genetic structure analysis. Molecular Ecology Resources, 12, 873–884. 10.1111/j.1755-0998.2012.03156.x 22639868

[ece34762-bib-0092] Rouse, J. W. , Haas, R. H. , Deering, D. W. , Schell, J. A. , & Harlan, J. C. (1974). Monitoring the vernal advancement of retrogradation of natural vegetation. Technical report, NASA/GSFC, Type III, Final Report, Greenbelt, MD, USA.

[ece34762-bib-0093] Rousset, F. (2008). GENEPOP’007: A complete re‐implementation of the GENEPOP software for Windows and Linux. Molecular Ecology Resources, 8, 103–106. 10.1111/j.1471-8286.2007.01931.x 21585727

[ece34762-bib-0094] Ruiz‐Lopez, M. J. , Barelli, C. , Rovero, F. , Roos, C. , Peterman, W. E. , & Ting, N. (2016). A novel landscape genetic approach demonstrates the effects of human disturbance on the Udzungwa red colobus monkey (*Procolobus gordonorum*). Heredity, 116, 167–176. 10.1038/hdy.2015.82 26374237 PMC4806883

[ece34762-bib-0095] Russo, I. M. , Sole, C. L. , Barbato, M. , von Bramann, U. , & Bruford, M. W. (2016). Landscape determinants of fine‐scale genetic structure of a small rodent in a heterogeneous landscape (Hluhluwe‐iMfolozi Park, South Africa). Scientific Reports, 6, 29168 10.1038/srep29168. 27406468 PMC4942783

[ece34762-bib-0096] Sawyer, S. C. , Epps, C. W. , & Brashares, J. S. (2011). Placing linkages among fragmented habitats: Do least‐cost models reflect how animals use landscapes? Journal of Applied Ecology, 48, 668–678. 10.1111/j.1365-2664.2011.01970.x

[ece34762-bib-0097] Scrucca, L. (2013). GA: A package for genetic algorithms in R. Journal of Statistical Software, 53, 1–37.

[ece34762-bib-0098] Shirk, A. J. , Landguth, E. L. , & Cushman, S. A. (2017). A comparison of individual‐based genetic distance metrics for landscape genetics. Molecular Ecology Resources, 17, 1308–1317. 10.1111/1755-0998.12684. 28449317

[ece34762-bib-0099] Sikes, R. S. & the Animal Care and Use Committee of the American Society of Mammalogists . (2016). Guidelines of the American Society of Mammalogists for the use of wild mammals in research and education. Journal of Mammalogy, 97, 663–688. 10.1093/jmammal/gyw078 29692469 PMC5909806

[ece34762-bib-0100] Soltz‐Herman, C. , & Valone, T. J. (2000). The effect of mammalian predator scent on the foraging behavior of *Dipodomys merriami* . Oikos, 91, 139–145. 10.1034/j.1600-0706.2000.910113.x

[ece34762-bib-0101] Spear, S. F. , Balkenhol, N. , Fortin, M.‐J. , McRae, B. H. , & Scribner, K. (2010). Use of resistance surfaces for landscape genetics studies: Considerations for parameterization and analysis. Molecular Ecology, 19, 3576–3591. 20723064 10.1111/j.1365-294X.2010.04657.x

[ece34762-bib-0102] Spear, S. F. , Cushman, S. A. , & McRae, B. H. (2015). Resistance surface modeling in landscape genetics In Balkenhol N., Cushman S. A., Storfer A. T., & Waits L. P. (Eds.), Landscape Genetics: Concepts, Methods, Applications (pp. 129–148). Chichester, UK: John Wiley & Sons Ltd.

[ece34762-bib-0103] Spear, S. F. , & Storfer, A. (2008). Landscape genetic structure of coastal tailed frogs (*Ascaphus truei*) in protected vs. managed forests. Molecular Ecology, 17, 4642–4656. 19140987 10.1111/j.1365-294X.2008.03952.x

[ece34762-bib-0104] Spear, S. F. , & Storfer, A. (2010). Anthropogenic and natural disturbance lead to differing patterns of gene flow in the Rocky Mountain tailed frog, *Ascaphus montanus* . Biological Conservation, 143, 778–786. 10.1016/j.biocon.2009.12.021

[ece34762-bib-0105] Springer, J. T. (2003). Home range size estimates based on number of relocations. Occas Wildlife Managem Papers, Univ Nebraska Kearney 14:1‐12.

[ece34762-bib-0106] Storfer, A. , Murphy, M. A. , Spear, S. F. , Holderegger, R. , & Waits, L. P. (2010). Landscape genetics: Where are we now? Molecular Ecology, 19, 3496–3514. 10.1111/j.1365-294X.2010.04691.x 20723061

[ece34762-bib-0107] Van Oosterhout, C. , Hutchinson, W. F. , Wills, D. P. M. , & Shipley, P. (2004). Micro‐checker: Software for identifying and correcting genotyping errors in microsatellite data. Molecular Ecology Notes, 4, 535–538. 10.1111/j.1471-8286.2004.00684.x

[ece34762-bib-0108] Van Strien, M. J. , Keller, D. , & Holderegger, R. (2012). A new analytical approach to landscape genetic modelling: Least‐cost transect analysis and linear mixed models. Molecular Ecology, 21, 4010–4023. 10.1111/j.1365-294X.2012.05687.x 22738667

[ece34762-bib-0109] VanEtten, J. (2017). R package Gdistance: Distances and Routes on Geographical Grids. R package version 1.2‐1. Retrieved from http://CRAN.R-project.org/package=gdistance

[ece34762-bib-0110] Waits, L. P. , Cushman, S. A. , & Spear, S. F. (2015). Applications of landscape genetics to connectivity research in terrestrial animals In Balkenhol N., Cushman S. A., Storfer A. T., & Waits L. P. (Eds.), Landscape Genetics: Concepts, Methods, Applications (pp. 199–219). West Sussex, UK: John Wiley & Sons Ltd.

[ece34762-bib-0111] Walsberg, G. E. (2000). Small mammals in hot deserts: Some generalizations revisited. BioScience, 50, 109–120. 10.1641/0006-3568(2000)050[0109:SMIHDS]2.3.CO;2

[ece34762-bib-0112] Waser, P. M. , Busch, J. D. , McCormick, C. R. , & DeWoody, J. A. (2006). Parentage analysis detects cryptic precapture dispersal in a philopatric rodent. Molecular Ecology, 15, 1929–1937. 10.1111/j.1365-294X.2006.02893.x 16689908

[ece34762-bib-0113] Wasserman, T. N. , Cushman, S. A. , Schwartz, M. K. , & Wallin, D. O. (2010). Spatial scaling and multi‐model inference in landscape genetics: *Martes americana* in northern Idaho. Landscape Ecology, 25, 1601–1612. 10.1007/s10980-010-9525-7

[ece34762-bib-0114] White, G. C. , & Garrot, R. A. (1990). Analysis of wildlife radio tracking data. San Diego, CA: Academic Press.

[ece34762-bib-0115] Whitford, W. G. (2002). Ecology of desert systems. San Diego, CA: Academic Press. An Elsevier Science Imprint

[ece34762-bib-0116] Worton, B. J. (1989). Kernel methods for estimating the utilization distribution in home range studies. Ecology, 1, 164–168. 10.2307/1938423

[ece34762-bib-0117] Zeller, K. A. , McGarigal, K. , & Whiteley, A. R. (2012). Estimating landscape resistance to movement: A review. Landscape Ecology, 27, 777–797. 10.1007/s10980-012-9737-0

[ece34762-bib-0118] Zeller, K. A. , Nijhawan, S. , Salom‐Pérez, R. , Potosme, S. H. , & Hines, J. E. (2011). Integrating occupancy modeling and interview data for corridor identification: A case study for jaguars in Nicaragua. Biological Conservation, 144, 892–901. 10.1016/j.biocon.2010.12.003

[ece34762-bib-0119] Zeng, Z. , & Brown, J. H. (1987). Population ecology of a desert rodent: *Dipodomys merriami* in the Chihuahuan desert. Ecology, 68, 1328–1340. 10.2307/1939217

