# Supplementary material for: Functional connectivity and home range inferred at a microgeographic landscape genetics scale in a desert‐dwelling rodent
Source: Ecol Evol. 2018 Dec 11;9(1):437–53. doi: 10.1002/ece3.4762 (PMC6342108; doi:10.1002/ece3.4762)
Supplement: Supplementary file 1 [file ECE3-9-437-s001.docx]

**Appendix**

**Material and methods**

**DNA extraction and microsatellite genotyping**

DNA was extracted from tissue samples with the ZR Genomic DNA-Tissue MiniPrep Kit (Zymo Research), following the manufacturer’s protocols. DNA quantity and quality were assessed with 1% agarose gels stained with GelRed (Biotium) and visualized with UV light. We tested 14 fluorescently labeled microsatellite primers developed for *D. spectabilis*: Ds1, Ds3, Ds19, Ds28, Ds30, Ds46 (Davis et al., 2000), DS92, DS98, DS107, DS108, DS109, DS163, DS222 and DS281 (Waser et al., 2006). Nine microsatellite primers were successfully amplified for *D. merriami* (Table S1 in the Appendix), following the thermocycling conditions given by the authors (Supplementary Material S1). All reactions were carried out in a 5 µL total volume containing 0.1 µL of Taq polymerase (Vivantis), 2% of BSA and ca. 30 ng of genomic DNA, and specific DNTPs, Cl_2_Mg, and primer concentrations (see Table S1 in the Appendix). Microsatellite products were run on an ABI Prism 3730xl and 3100 Genetic Analyzer (Applied Biosystems), with ROX-500 as internal size standard and allele size determined with the software GeneMarker v2.2.0 (SoftGenetics). We included negative controls in all runs and multiple samples were sized at least twice to assure reproducibility and correct readings.

**References**

Davis, C., Keane, B., Swanson, B., Loew, S., Waser, P. M., Strobeck, C., Fleischer, R. C. 2000. Characterization of microsatellite loci in bannertailed and giant kangaroo rats, *Dipodomys spectabilis* and *Dipodomys ingens*. *Molecular Ecology* 9:642-644.

Waser, P. M., Busch, J. D., McCormick, C. R., DeWoody, J. A. 2006. Parentage analysis detects cryptic precapture dispersal in a philopatric rodent. Molecular Ecology, 15:1929-1937.

**Table S1.** Microsatellite loci used in the present study with *Dipodomys merriami*, based on those from Davis et al. (2000) (Ds1, Ds3, Ds19, Ds30, Ds46) and Waser et al. (2006) (DS98, DS107, DS109, DS163).

PCR conditions*

Locus Primer sequence (5'-3') Motif Ta Dye Range MgCl_2_ dNTPs Primers *Taq*

(°C) (mM) (mM) (µM)

Ds1 F: GATCAACCACCCAGCTCTAT di 54 FAM 138-234 1.0 0.12 0.16 0.1 uL

R: GCAAAGCCCTGAGTTCAAAAG

Ds3 F: TCAAGCTCCAGGACAGCACAAG di 54 HEX 171-205 1.0 0.12 0.16 0.1 uL

R: GTTTCCATTGATGCCCAGCAGATTT

Ds19 F: ATCCTCAGAACCTTCATTCA di 54 FAM 98-128 2.0 0.12 0.16 0.1 uL

R: AATCTATGAGTGAGCCAACAG

Ds30 F: ATCCTTCCTCCCAATGTTGTAG di 54 FAM 241-273 1.0 0.12 0.16 0.1 uL

R: GTTTCCCAGGCAGAAAAGTTTATGATA

Ds46 F: CTAATCACCGAGCCAAA di 54 HEX 272-298 3.5 0.12 0.16 0.1 uL

R: GTTTATCAACTATAAAAATCATAGAAAA

DS98 F: GCCTATCTGCCCATCTACCA tetra 50 HEX 204-248 1.9 0.25 0.5 0.1 uL

R: GGCATTCTGAAAGCGAGAAG

DS107 F: TGTTCTCTTTACAGGAACTG di 47 FAM 131-165 2.0 0.25 0.4 0.1 uL

R: CTGAGCTAATATGTGAGGTA

DS109 F: GCTCCTAGGTAGGATCATTC tetra 47 FAM 122 1.5 0.25 0.4 0.1 uL

R: CAGAAGCACTCTGAATCATTG

DS163 F: CATGCTCAATGCAATCGTCT di 51 HEX 209-235 1.88 0.25 0.5 0.1 uL

R: TGGATTCTCAACCTGCTCCT

*All reactions included a 2% BSA

**Table S2.** Genetic diversity of *Dipodomys merriami* individuals from the Mapimí Biosphere Reserve, Mexico, estimated as number of alleles (*A*), number of effective alleles (*Ae*), unbiased observed heterozygosity (*Ho*), expected heterozygosity (*He*), and (*F*IS).

| Locus | *A* | *Ae* | *Ho* | *He* (Nei) | *F*IS |  |
| --- | --- | --- | --- | --- | --- | --- |
| Ds1 | 30 | 17.718 | 0.763 | 0.943 | 0.191 |  |
| Ds3 | 12 | 4.933 | 0.566 | 0.806 | 0.290 |  |
| Ds19 | 13 | 9.139 | 0.408 | 0.862 | 0.542 |  |
| Ds30 | 15 | 5.230 | 0.789 | 0.814 | 0.024 |  |
| Ds46 | 14 | 5.663 | 0.447 | 0.837 | 0.457 |  |
| DS98 | 12 | 9.531 | 0.763 | 0.895 | 0.147 |  |
| DS107 | 17 | 9.848 | 0.895 | 0.895 | 0.004 |  |
| DS163 | 8 | 3.205 | 0.618 | 0.691 | 0.101 |  |
| Mean | 15.125 | 8.158 | 0.656 | 0.843 | 0.220 |  |
| s.d. | 6.556 | 4.580 | 0.174 | 0.077 | 0.196 |  |

**Table S3.** Parameter estimates from mixed effects models fit to optimized resistance surfaces. Beta, standard error (SE) and t-value estimates are from model fit by restricted maximum likelihood. Models that performed better than distance alone are in bold.

| **Resistance surface** | **Parameter** | ***ß*** | **SE** | **t - value** |
| --- | --- | --- | --- | --- |
| **Elevation** | **Intercept** | **0.808** | **0.008** | **100.401** |
|  | **elevation** | **0.004** | **0.004** | **1.248** |
|  |  |  |  |  |
| Distance | Intercept | 0.808 | 0.008 | 100.293 |
|  | distance | 0.000 | 0.003 | 0.171 |
|  |  |  |  |  |
| **NDVI** | **Intercept** | **0.808** | **0.008** | **101.495** |
|  | **fondondvi2** | **0.010** | **0.005** | **1.963** |
|  |  |  |  |  |
| **Humidity** | **Intercept** | **0.808** | **0.008** | **100.209** |
|  | **humidity** | **0.009** | **0.005** | **1.713** |
|  |  |  |  |  |
| Feature | Intercept | 0.808 | 0.008 | 99.872 |
|  | maprecorte | -0.001 | 0.005 | -0.265 |
|  |  |  |  |  |
| **Temperature** | **Intercept** | **0.808** | **0.008** | **99.598** |
|  | **temperature** | **0.004** | **0.004** | **1.089** |

**Table S4.** Spearman correlation test between surfaces with higher percentage than distance alone. Surfaces with a small to medium correlation are indicated with an asterisk. Spearman correlation coefficient (ρ).

| **Surface 1** | **Surface 2** | **ρ** |
| --- | --- | --- |
| NDVI | Humidity | -0.1108* |
| NDVI | Temperature | -0.0069* |
| NDVI | Elevation | -0.4006 |
| Humidity | Temperature | 0.3786 |
| Humidity | Elevation | -0.3477 |
| Temperature | Elevation | -0.2218* |

**Table S5.** Parameter estimates from mixed effects models fit to composite resistance surfaces. Beta, standard error (SE) and t-value estimates are from model fit by restricted maximum likelihood.

**Resistance surface Layers Parameter ß SE t - value**

Combination 1 NDVI, Humidity Intercept 0.808 0.008 100.209

fondondvi2.humidity 0.009 0.005 1.720

Combination 2 NDVI, Temperature Intercept 0.808 0.008 99.645

fondondvi2.

temperature 0.004 0.004 1.086

Combination 3 Temperature, Intercept 0.808 0.008 100.293

Elevation temperature.

elevation 0.000 0.003 0.171

Combination 4 NDVI, Humidity, Intercept 0.808 0.008 100.294

Temperature, fondondvi2.humidity.

Elevation temperature.

elevation 0.000 0.003 0.169


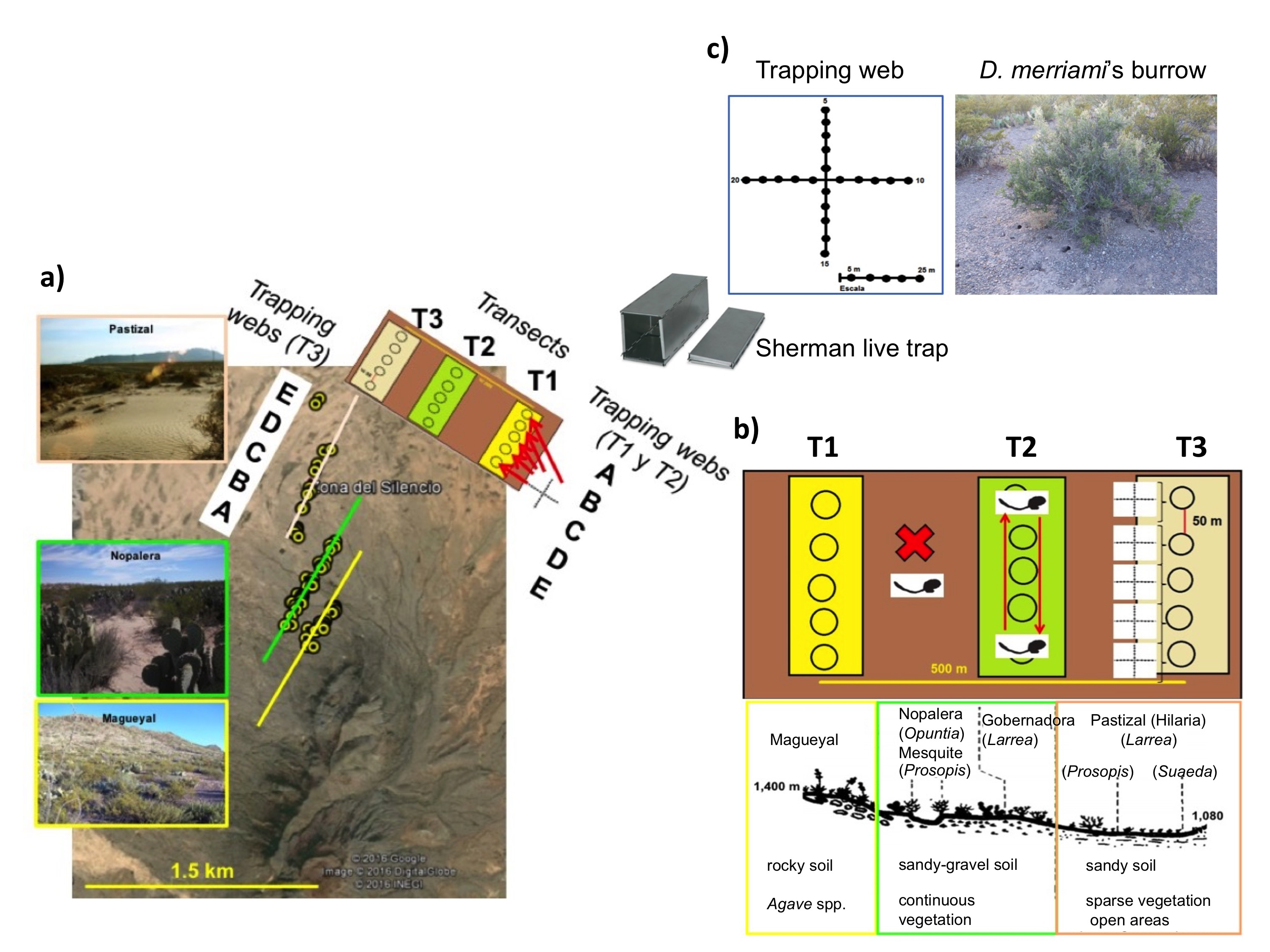


**Figure S1** Sampling strategy used for the landscape genetics study of *Dipodomys merriami* individuals on the Mapimí Biosphere Reserve, Mexico, based on a modification of the trapping web sampling method (Anderson *et al.* 1983; Serrano 1987). **a**) Google Earth image of the study site depicting the location of the three transects, **b**) the three vegetation transects are shown, which were located along different vegetation/soil/elevation zones: Magueyal (T1), Nopalera (T2), Pastizal (T3). The transects were separated by approximately 200 m, where five trapping webs were set per transect, separated 50 m from each other. Red cross depicts no movement of individuals between transects (where no vegetation is present), while red arrows (T2), hypothesizes movement along the transect. **c**) Each web consisted of four 25 m lines radiating from a central point (often a *Dipodomys merriami*’s burrow), where Sherman live traps (7.6 x 7.6 x 33 cm) were placed along each line, separated by 5 m; additionally, two more traps were placed at one meter from the burrow entrance.

Serrano, V. (1987). Las comunidades de roedores desertícolas del Bolsón de Mapimí, Durango. *Acta Zoológica Mexicana*, *20*, 1-22; Anderson, D. R., Burnham, K. P., White, G. C., & Otis, D. L. (1983). Density estimation of small-mammal populations using a trapping web and distance sampling methods. *Ecology*, *64*, 674–680.


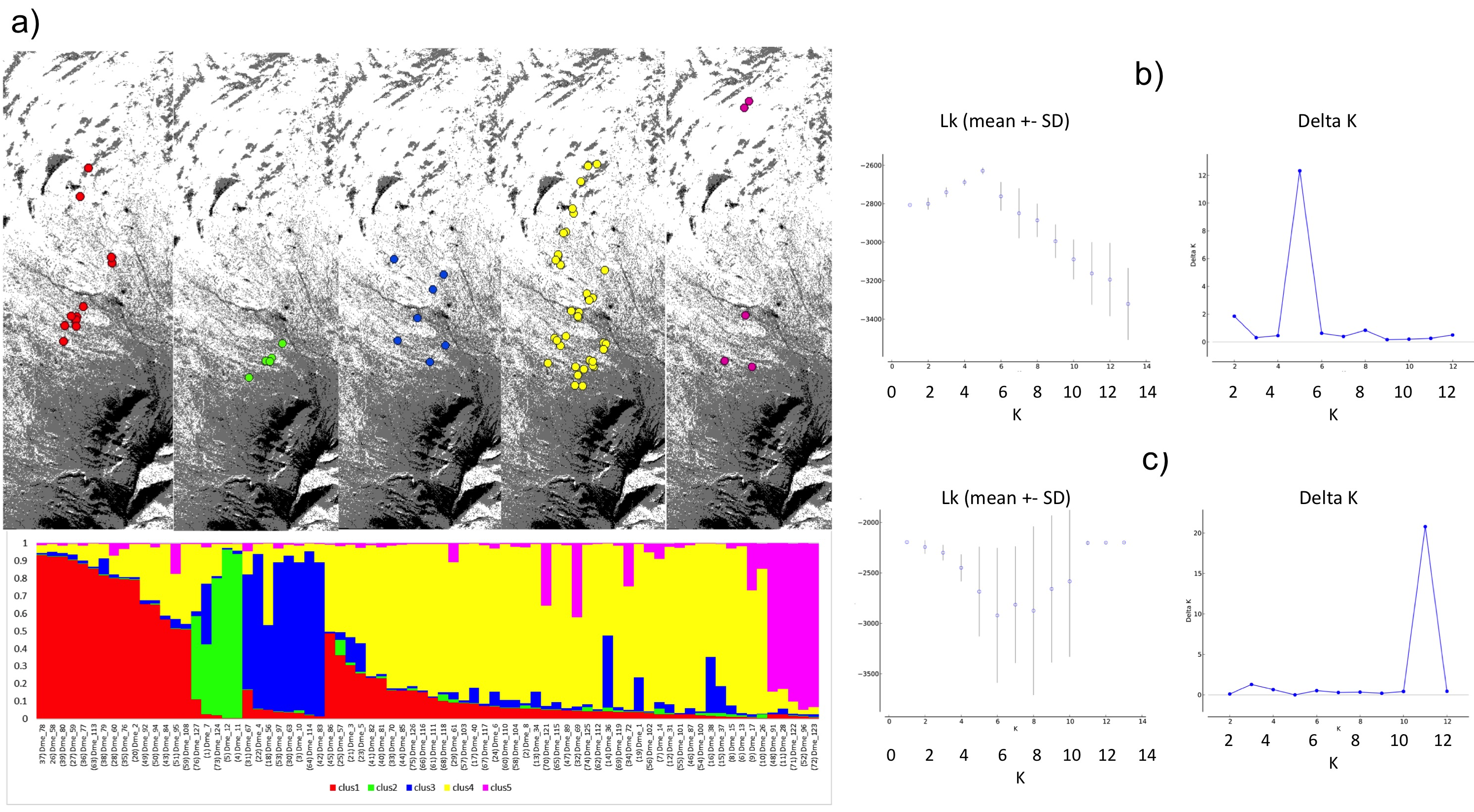


**Figure S2** Structure results for *Dipodomys merriami* on the Mapimí Biosphere Reserve, Mexico: **a**) Bar plot showing individual membership to one of the five (*K*=5) genetic clusters (each cluster is represented by a different color and each labeled vertical line depicts an individual); spatial distribution of individuals by cluster is also shown (colors correspond to that of each cluster). Mean log likelihood over 20 runs (error bars = standard deviations) and ∆K for each *K* for **b**) for the full dataset (n=76) and **c**) unrelated dataset (n=59).


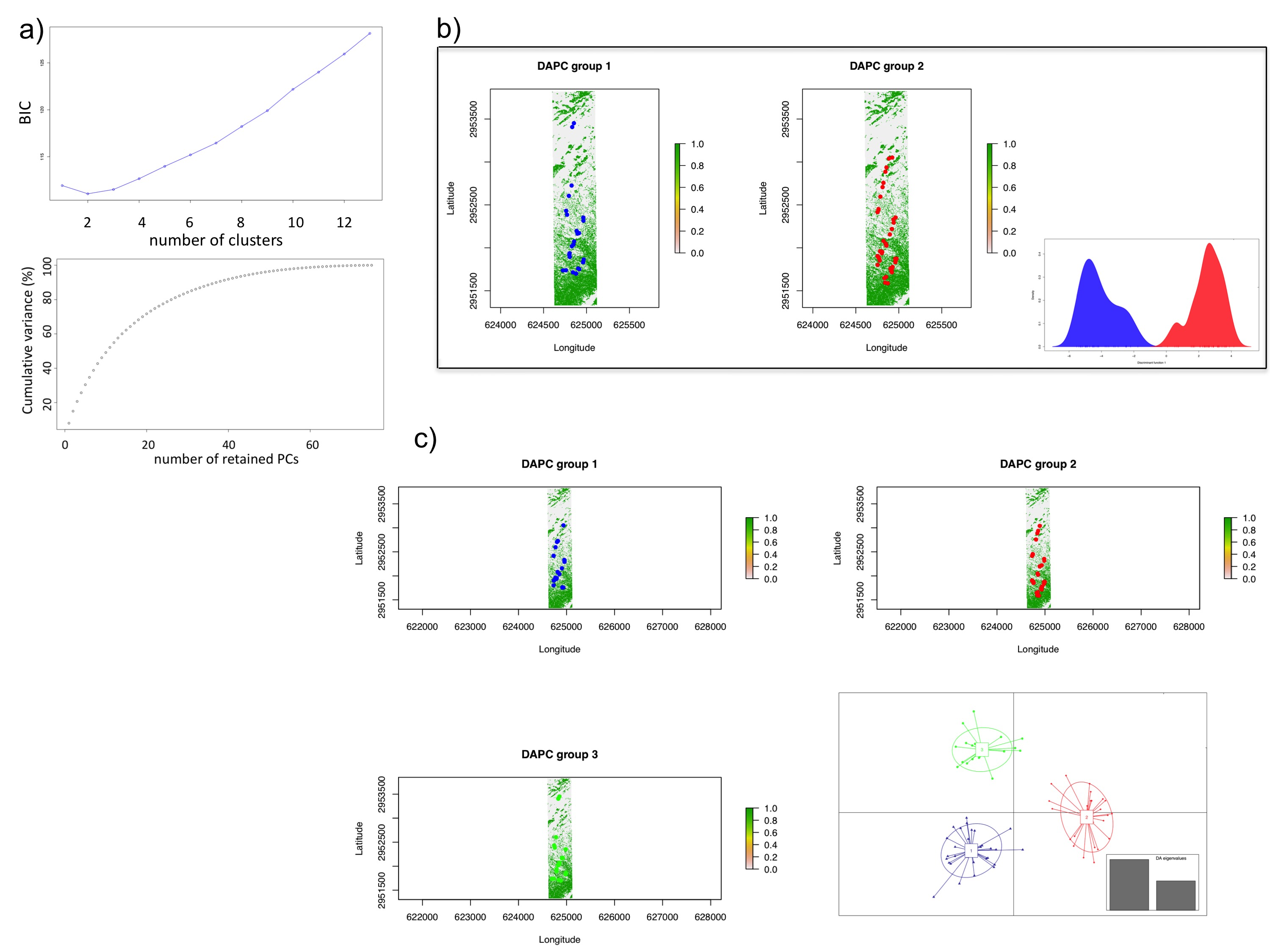


**Figure S3** Discriminant Analysis of Principal Components (DAPC) for *Dipodomys merriami* from the Mapimí Biosphere Reserve, Mexico, based on the complete dataset (n=76). **a**) BIC values for each *K* value tested (top), based on the selection of PCs that encompassed the highest variance (bottom). Spatial distribution of genetic clusters and scatter plot for the different number of clusters **b**) *K*=2 and **c**) *K*=3.


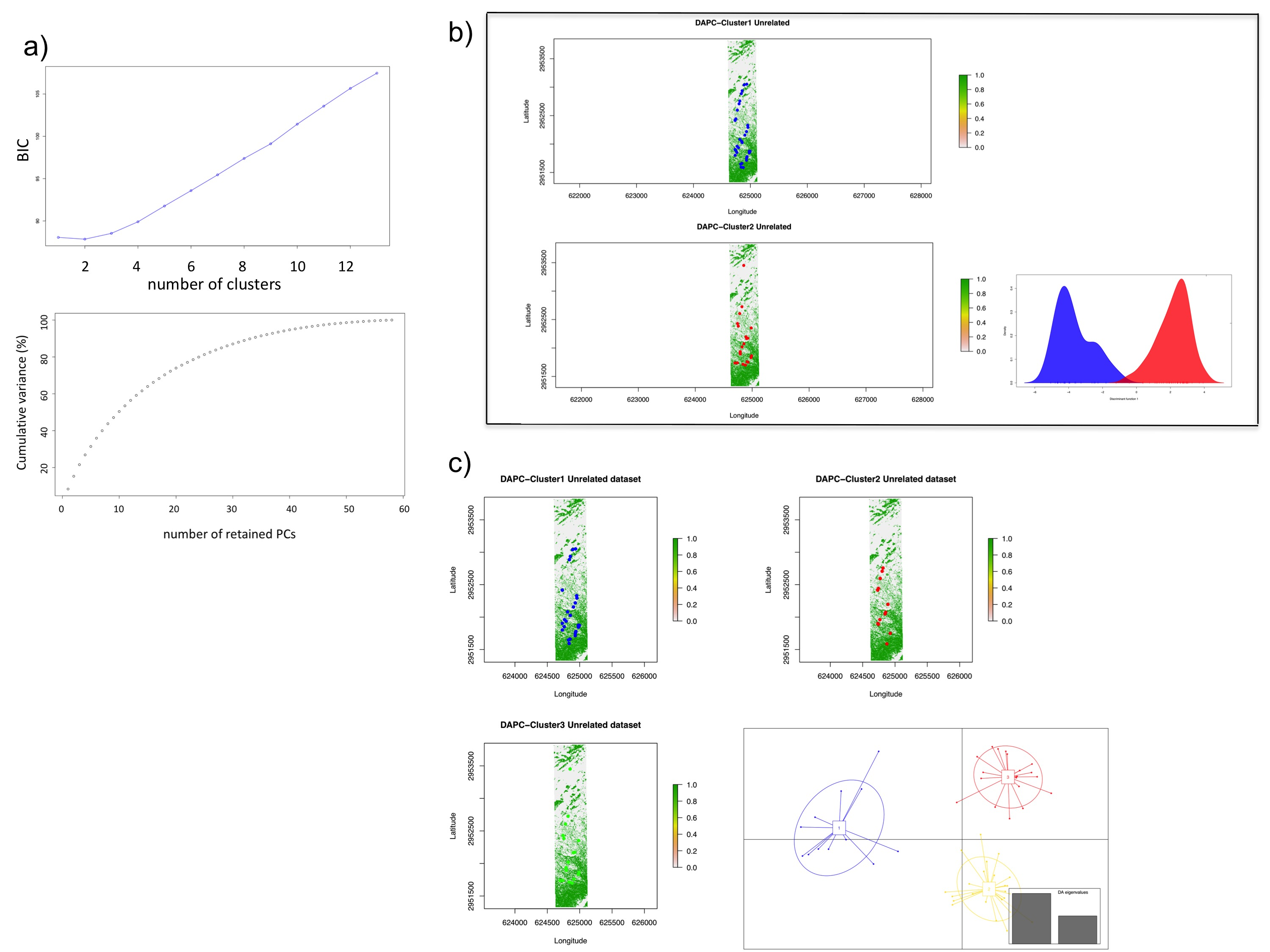


**Figure S4** Discriminant Analysis of Principal Components (DAPC) for *Dipodomys merriami* from the Mapimí Biosphere Reserve, Mexico, based on the unrelated dataset (n=59). **a**) BIC values for each *K* value tested (top), based on the selection of PCs that encompassed the highest variance (bottom). Spatial distribution of genetic clusters and scatter plot for the different number of clusters **b**) *K*=2 and **c**) *K*=3.


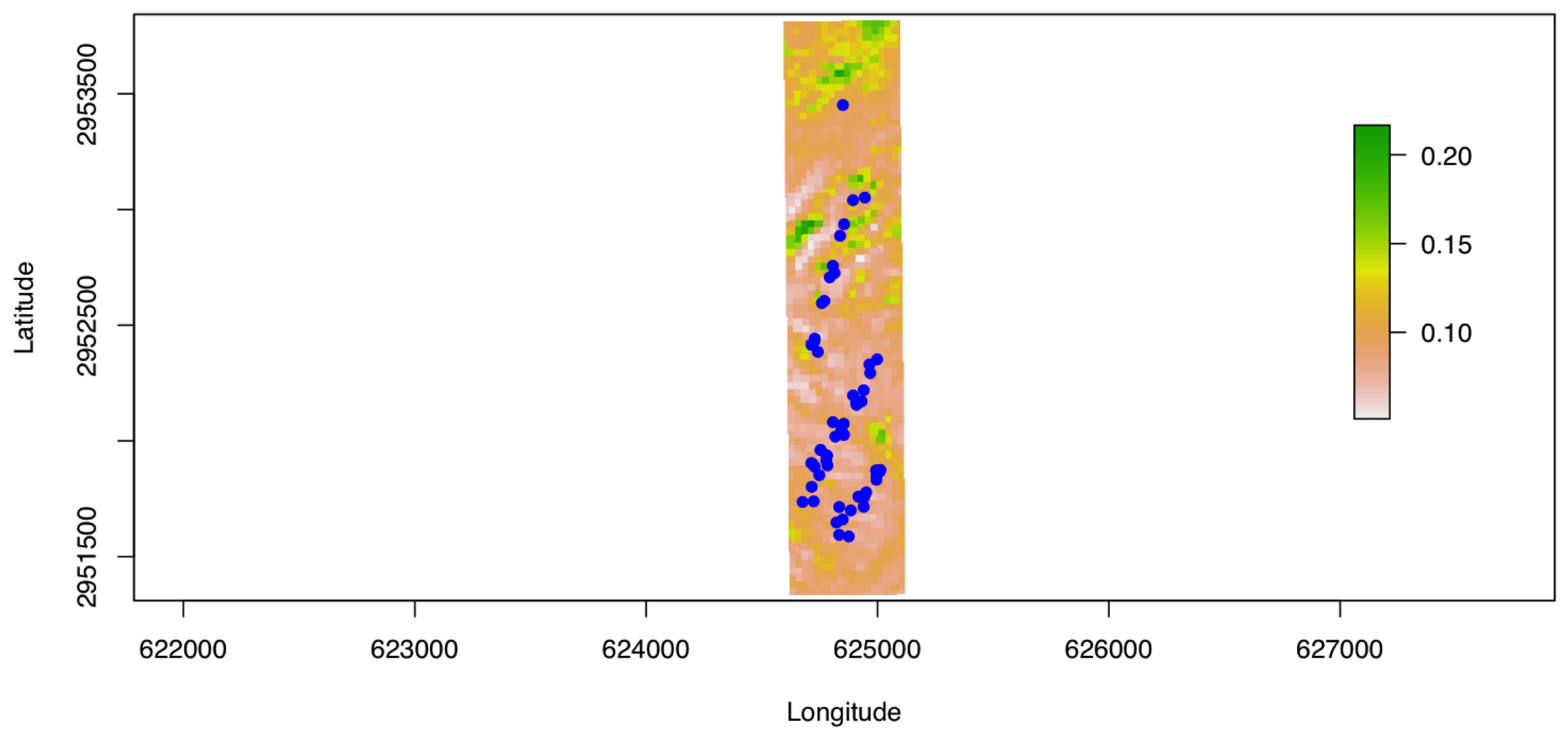


**Figure S5**  Sampling sites of individuals of *Dipodomys merriami* from the Mapimí Biosphere Reserve, Mexico, used for the landscape genetics analyses depicted by blue dots, based on the unrelated dataset (n=59). These are plotted on the NDVI surface generated for the study area.


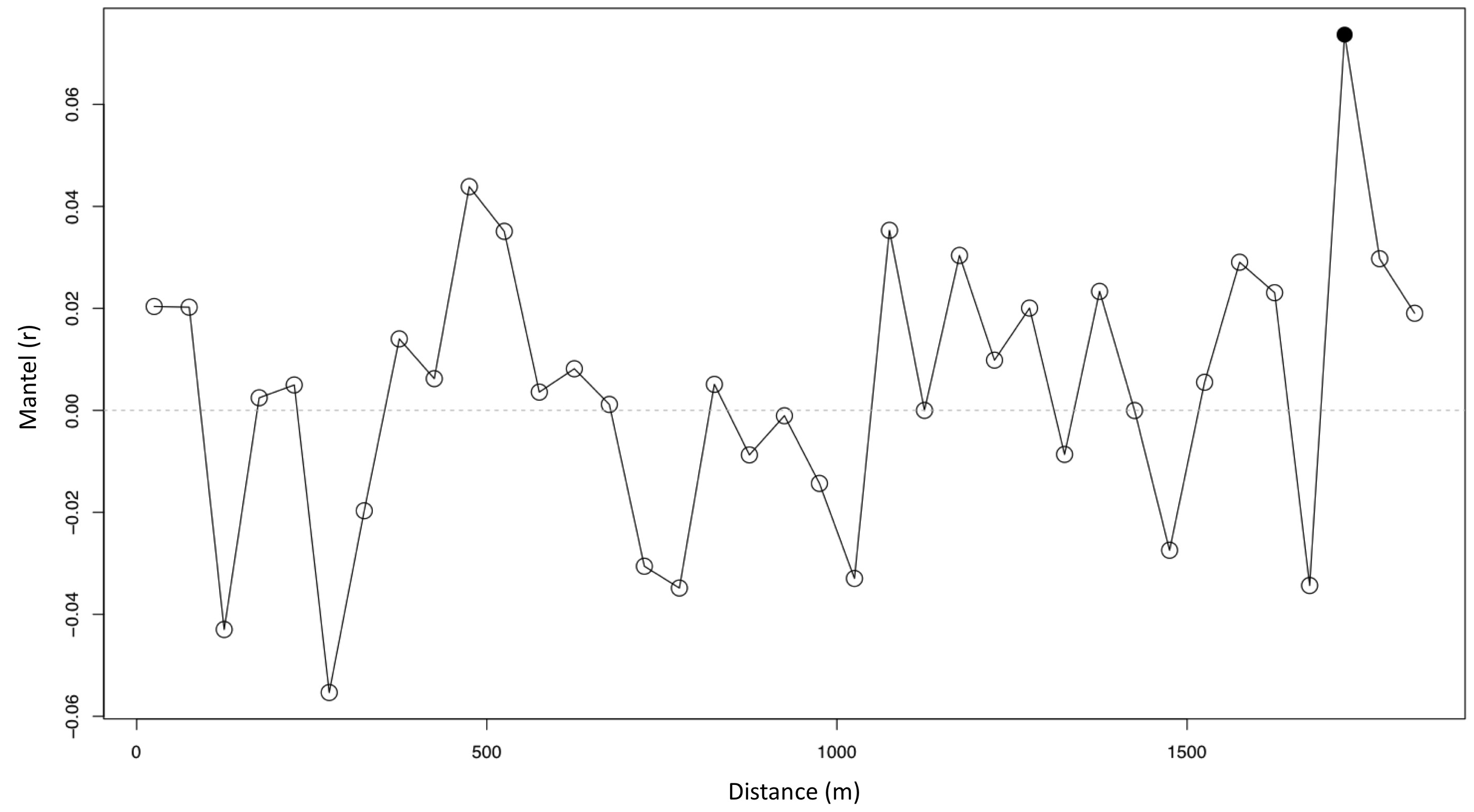


**Figure S6**  Mantel correlogram for the geographic (Euclidean) and genetic (*D*_PS_) distances calculated at 50 m distance classes for *Dipodomys merriami* from the Mapimí Biosphere Reserve, Mexico. A significant value (Spearman; *r* = 0.073; *p*<0.05) shown in black corresponds to a distance up to 1,750 m.

**R scripts used for the optimization analyses in the present study**

**SCRIPT 1**

# Script for optimizing Surfaces contained in a Rasterstack object

# Surfaces are optimized ONE BY ONE

# This script can be modified for using a different unit level (i.e. Population) or a different genetic distance metric

# 1. Load packages

library(ResistanceGA)

library(ggplot2)

library(gdistance)

library(gstudio)

library(PopGenReport)

# 2. Import landscape data

load("./landscapegenetics_data/landscape_surfaces.rda") # Landscape surfaces (Rasterstack obkect)

# 3. Estimate *D*_PS_ genetic distances and get spatial location by individual

# 3.1 *D*_PS_ corresponds to (1-Dps) (Bowcock et al. 1994)

dme <- read.genetable("./Data/dmerriami_dataset.csv", ind=1, pop=2, lat=3,

long=4, x=5, y=6, other.min = 7,

other.max = 9, oneColPerAll = TRUE) # This line will depend on your own dataset. Verify the number and names of your columns.

dme # genind object

Ps <- propShared(dme)

Dps <- (1-Ps)

Dps # matrix of pairwise genetic distances

# 3.2 Load in points (SpatialPoints object)

dipo <- read_population("./Data/dmerriami_dataset.csv", type="column",locus.columns=10:25) # This line will depend on your own dataset. Verify the number and names of your columns.

coords <- strata_coordinates( dipo, stratum="ind", longitude="x", latitude="y" )

pts <- SpatialPoints( coords[,2:3], proj4string=CRS("+proj=utm +zone=13 +ellps=WGS84 +datum=WGS84 +units=m +no_defs"))

# 4. Create a directory for saving results and a subdirectory for every run

dir.create("./ResistanceGA_Results") # Choose a name

dir.create(file.path("./ResistanceGA_Results/","run1_date")) # I recommend to add the date of each run for tracking your progress (e.g. "run1_DDMMYYYY")

# 4.1. Selecting Directory to write .asc files and results. RUN THIS LINE FOR EVERY OPTIMIZATION PROCESS!

write.dir <- "./ResistanceGA_Results/run1_date/"

# 5. Getting ready for Optimization!

# NOTE: This is the INITIAL OPTIMIZATION of surfaces in the rasterstack object, one by one, using DEFAULT parameters and AIC method.

GA.inputs_All <- GA.prep(method = "AIC",

ASCII.dir = r.stack, # the Rasterstack object

Results.dir = write.dir, # Check this folder each time

min.cat = 0, # If you have categorical and/or binary surfaces, specify this value. Otherwise, omit it!

select.trans = list("A",

"A",

"A",

"A",

NA), # According to the order of each surface in the rasterstack object, select "A" for evaluate All transformations. NA indicates the 5th position of categorical surface in the Rasterstack object. No transformation is applied to this surface.

parallel = 4)

gdist.inputs <- gdist.prep(n.Pops = length(pts), # Spatial points object

samples = pts,

response = lower(Dps), # Genetic distance matrix

method = 'commuteDistance') # Least-Cost or Circuit-Theory

# 6. Run optimization

allsurfaces_date <- SS_optim(gdist.inputs = gdist.inputs, GA.inputs = GA.inputs_All)

# 7. Saving results

# This step is fundamental for bootstrap analysis! See next Script.

save(allsurfaces_date, file = "allsurfaces_date.rda")

**SCRIPT 2**

# Script for conducting bootstrap analysis from previous optimized surfaces

library(ResistanceGA)

library(PopGenReport)

# 1. Load Optimization surfaces results (previous script)

load("./ResistanceGA_Results/run1_date/ allsurfaces_date.rda")

# 2. Extract relevant components from optimization outputs

# Make a list of cost/resistance distance matrices

mat.list <- allsurfaces_date$cd

k <- rbind(allsurfaces_date$k)

# 3. Load or calculate response variable (genetic distance) as matrix. In this case, we have already calculated Dps (previous script, step 3.1).

# Remove col and row names

colnames(Dps) <- NULL

rownames(Dps) <- NULL

response <- Dps

# 4. Run bootstrap

AIC.boot <- Resist.boot(mod.names = names(mat.list), dist.mat = mat.list,

n.parameters = k[,2],

sample.prop = 0.75,

iters = 10000,

obs = 59, # This value will depend on the number of individuals/populations

genetic.mat = response)

AIC.boot

# 5. Saving bootstrap results

save(AIC.boot, file = "AICboot_date.rda")
